# Genetic effects and causal association analyses of 14 common conditions/diseases in multimorbidity patterns

**DOI:** 10.1371/journal.pone.0300740

**Published:** 2024-05-16

**Authors:** Ting Fu, Yi-Qun Yang, Chang-Hua Tang, Pei He, Shu-Feng Lei

**Affiliations:** 1 Collaborative Innovation Center for Bone and Immunology between Sihong Hospital and Soochow University, Center for Genetic Epidemiology and Genomics, School of Public Health, Suzhou Medical College of Soochow University, Suzhou, Jiangsu P. R. China; 2 Department of Orthopedics, Sihong Hospital, Suzhou, Jiangsu, P. R. China; 3 Jiangsu Key Laboratory of Preventive and Translational Medicine for Geriatric Diseases, Soochow University, Suzhou, Jiangsu, P. R. China; 4 Changzhou Geriatric Hospital Affiliated to Soochow University, Changzhou, China; State University of New York Upstate Medical University, UNITED STATES

## Abstract

**Background:**

Multimorbidity has become an important health challenge in the aging population. Accumulated evidence has shown that multimorbidity has complex association patterns, but the further mechanisms underlying the association patterns are largely unknown.

**Methods:**

Summary statistics of 14 conditions/diseases were available from the genome-wide association study (GWAS). Linkage disequilibrium score regression analysis (LDSC) was applied to estimate the genetic correlations. Pleiotropic SNPs between two genetically correlated traits were detected using pleiotropic analysis under the composite null hypothesis (PLACO). PLACO-identified SNPs were mapped to genes by Functional Mapping and Annotation of Genome-Wide Association Studies (FUMA), and gene set enrichment analysis and tissue differential expression were performed for the pleiotropic genes. Two-sample Mendelian randomization analyses assessed the bidirectional causality between conditions/diseases.

**Results:**

LDSC analyses revealed the genetic correlations for 20 pairs based on different two-disease combinations of 14 conditions/diseases, and genetic correlations for 10 pairs were significant after Bonferroni adjustment (*P*<0.05/91 = 5.49E-04). Significant pleiotropic SNPs were detected for 11 pairs of correlated conditions/diseases. The corresponding pleiotropic genes were differentially expressed in the brain, nerves, heart, and blood vessels and enriched in gluconeogenesis and drug metabolism, biotransformation, and neurons. Comprehensive causal analyses showed strong causality between hypertension, stroke, and high cholesterol, which drive the development of multiple diseases.

**Conclusions:**

This study highlighted the complex mechanisms underlying the association patterns that include the shared genetic components and causal effects among the 14 conditions/diseases. These findings have important implications for guiding the early diagnosis, management, and treatment of comorbidities.

## Introduction

Multimorbidity is a coexistence state of two or more medical conditions/diseases [1, 2], which commonly occurs in older adults. The estimated prevalence of multimorbidity was 67.8% in older adults aged 65 years and older by 2035 in the UK [3]. These chronic diseases mainly include long-term mental conditions/diseases, physical noncommunicable diseases of long duration, and long-term chronic diseases of the musculoskeletal system. Comorbidity substantially increases the medical and socioeconomic burden. Especially during the COVID-19 pandemic, individuals with multimorbidity have been at higher risk of infection and adverse outcomes [4].

The pathogenesis underlying the occurrence of multimorbidity is probably related to aging, heredity, and other factors (e.g., socioeconomic deprivation) [5]. In the comorbidity patterns, the correlations among the diseases are complex, and some diseases may be the driving factors of other diseases. For example, some chronic diseases contribute to the progression of other conditions/diseases, with slight clinical symptoms and undetectable symptoms, especially for older persons [6]. Diabetes and hypertension are the most common chronic diseases in Spanish people aged over 65 years with dementia [7]. In the observational study, Bendayan et al. ranked 14 common diseases to older adults in descending order of prevalence, with hypertension, heart disease, depression, and asthma [8]. A study that included 0.5 billion Chinese adults has identified four multimorbidity patterns, including cardiometabolic multimorbidity (diabetes, stroke and hypertension), cancer, psychiatric problems (neurasthenia) and arthritis [9]. A review study suggests that depression, hypertension, diabetes, arthritis and asthma can easily co-exist with other diseases [10]. However, in traditional cohort studies, the analysis of the association between diseases is easily affected by many confounding factors that are difficult to control or unknown, leading to unreliable results. For example, Poblador-Plou et al. studied multimorbidity patterns in people over 65 years of age in Spain [7]. They found that diabetes and hypertension were the most common chronic diseases in people with dementia. However, when they identified multimorbidity patterns, the patterns associated with dementia did not include diabetes or hypertension, but include Parkinson’s disease, osteoporosis, thyroid disease, anxiety, and neurosis. Therefore, in multimorbidity patterns, the complex correlation patterns and the underlying mechanisms for the correlations are still largely unknown.

Therefore, this study selected 14 conditions/diseases that were the main diseases experienced by middle and older age individuals and the major sources of long-standing illnesses for people aged 65 years and older in other national surveys (details can be found in https://www.elsa-project.ac.uk/data-and-documentation). We performed systemic analyses (e.g., linkage disequilibrium score regression (LDSC) [11], the pleiotropic analysis method under the composite original hypothesis (PLACO) [12], and mapping and functional annotation of pleiotropic SNPs were conducted by FUMA) [13] to detect the genetic correlations and the underlying pleiotropic genes and the driving conditions/diseases by inferring the causal relations (Mendelian randomization analysis) [14] among 14 conditions/diseases. Understanding the genetic components shared by them and the causal association patterns is essential for elucidating phenotypic relationships and disease pathology, which could help to provide basic data for the prevention, diagnosis, and treatment of these conditions/diseases.

## Methods and materials

### GWAS summary statistics for 14 conditions/diseases

Following the selection of the conditions/diseases in the English Longitudinal Study of Aging (ELSA) [8]. The study also selected the fourteen conditions/diseases, including hypertension, type 2 diabetes, cancer, chronic obstructive pulmonary disease, heart problems, stroke, psychiatric problems (mental health problems ever diagnosed by a professional: Anxiety, nerves or generalized anxiety disorder), arthritis, asthma, high cholesterol, cataracts, Parkinson’s disease, hip fracture, and depression. There are no ethical issues involved in this study and "ethical approval and consent to participate" is not applicable. The statistics of 14 conditions/diseases were downloaded from the GWAS **(Table 1)**.

**Table 1:** Basic characteristics of the 14 studied GWASs.

Trait	N	Ncase/Ncontrol	Year	Population	Source (GWAS ID)
Hypertension	463,010	54,358/408,652	2018	European	ukb-b-12493
T2D^a^	433,540	77,418/356,122	2018	European	ebi-a-GCST010118
Cancer	56,637	17,131/39,506	2021	European	GCST90086048
COPD^b^	462,933	1,605/461,328	2018	European	ukb-b-13447
Heart problem	462,933	1,486/461,447	2018	European	ukb-b-8315
Stroke	446,696	40,585/406,111	2018	European	ebi-a-GCST006906
Psychiatric problems	117,751	16,730/101,021	2018	European	ukb-d-20544_15
Arthritis	463,010	1,477/461,533	2018	European	ukb-b-11045
Asthma	337,159	39,049/298,110	2017	European	ukb-a-66
High cholesterol	462,933	56,753/406,180	2018	European	ukb-b-10912
Cataract	150,462	14,254/136,388	2018	European	ukb-b-8329
Parkinson’s disease	482,730	33,674/449,056	2019	European	ieu-b-7
Hip fracture	456,348	1,930/454,418	2021	European	GCST90044632
Depression	180,866	105,739/16,471	2016	European	ebi-a-GCST003769

Note: ^#^GWASs: Genome-wide association studies; N is the total sample size of GWASs; Ncase is the number of case groups; Ncontrol is the number of control groups; T2D^a^: type 2 diabetes; COPD^b^: chronic obstructive pulmonary disease. Psychiatric problems: Mental health problems ever diagnosed by a professional: Anxiety, nerves or generalized anxiety disorder. Hypertension, T2D, COPD, heart problem, stroke, psychiatric problem, arthritis, asthma, high cholesterol, cataract, Parkinson’s disease and depression were found from https://gwas.mrcieu.ac.uk; cancer and hip fracture were found from https://www.ebi.ac.uk/gwas/downloads/summary-statistics.

#### Genetic correlation analysis for each pair of condition/disease–LDSC

LDSC can estimate heritability and confounding bias between SNPs [15], and its principle and algorithm are well described [16]. We assessed the genetic correlation between two phenotypes by using LDSC (https://github.com/bulik/ldsc). LD scores could be calculated from the European samples in the 1000 Genomes Project as a reference panel [17]. Quality controls were implemented on the SNPs before LDSC analysis to filter out unqualified SNPs (e.g., nonbiallelic SNPs, stranded-ambiguous SNPs, duplicate SNPs, SNPs without rs numbers, SNPs with minor allele frequencies<0.01, SNPs located in the major histocompatibility complex (MHC) region with a complex LD structure (HLA region markers) and SNPs that were not in or whose alleles did not match Phase 3 of the 1000 Genomes Project) [18].

#### Pleiotropic analysis under the composite hypothesis

PLACO is a novel approach for investigating the presence of pleiotropic loci between different traits [19]. It utilizes genotype-phenotype association statistics at the aggregate level to identify pleiotropic loci [20]. We computed the squares of Z scores for each variant. Then, we calculated the correlation matrix of Z to consider the potential correlations for different diseases. To test the hypothesis of no pleiotropy, a level-α intersection-union test (IUT) was used: a composite null hypothesis *H*_0_: *H*_00_∪*H*_01_∪*H*_02,_ while the alternative hypothesis is *H*_1._ *H*_1_ is represented further as:

$$H_{1}:\;H_{00}^{c}\cap H_{01}^{c}\cap H_{02}^{c},\;\mathrm{where}$$

$$H_{00}:\;\beta_{\mathrm{trait}1}=\beta_{\mathrm{trait}2}=0;\;H_{01}:\;\beta_{\mathrm{trait}1}=0,\;\beta_{\mathrm{trait}2}\neq 0\;\mathrm{and}\;H_{02}:\;\beta_{\mathrm{trait}1}\neq 0,\;\beta_{\mathrm{trait}2}=0.$$

hc stands for the complement of *H*. *β* represents the effect size of the phenotype. Then, IUT tests the maximum P value of H_0_ and H_1_ as the final P value, which is replaced by the following asymptotic approximation:

$$P_{Z_{trait1}\;Z_{trait2}}=F(\frac{z_{trait1}\;z_{trait2}}{\sqrt{Var(z_{trait1})}})+F(\frac{z_{trait1}\;z_{trait2}}{\sqrt{Var(z_{trait2})}})-F(z_{trait1}\;z_{trait2})$$

z_trait1_ and z_trait2_ represent the observed Z scores for two conditions/diseases at a given genetic variant. F(y) is the two-sided tail probability of the normal product distribution for y. Var(x) is the expected value of the squared deviation of x from the mean. PLACO was performed by using the R package “PLACO”.

### The functional annotation of pleiotropic SNPs

Afterward, we functionally mapped and annotated the PLACO-screened pleiotropic SNPs using the online tool Functional Mapping and Annotation (FUMA, https://fuma.ctglab.nl/.) [13]. Independent significant SNPs were identified according to the default thresholds of *P*<5E-08 and r^2^<0.6 in FUMA. These SNPs are further represented by lead SNPs, which are a subset of the independent significant SNPs if the SNP had r^2^< 0.1. Then, we used positional mapping of deleterious coding SNPs and expression quantitative trait loci (eQTLs) to map independent significant SNPs to genes. SNPs were mapped to certain genes if the combined annotation-dependent depletion (CADD) score was >12.37 [13, 20]; otherwise, they were filtered. For eQTL mapping, all independent significant SNPs in LD were mapped to eQTLs in gene tissue expression (GTEx) v8 tissue types.

Finally, gene set enrichment analysis (GSEA) was undertaken to test the possible biological mechanisms of pleiotropic genes. Hypergeometric tests are conducted to assess the possible biological functions of genes of interest. A series of pathway enrichment analyses, including the Molecular Signatures Database (MSigDB) [21] and KEGG [22], were used. The expression (transcripts per million) of prioritized genes in different tissues was estimated from GTEx v8 following winsorization at 50 and log2 transformation [23]. The mRNA expression data were first normalized, and then, for each gene, Student’s two-sided t test was performed per tissue against all other tissues. The fold change (FC) of the gene expression level was utilized to represent the differential expression between different tissues. The adjusted p value < 0.05 (*P*bon<0.05) and absolute log fold change 0.58 were defined as a DEG set in a given tissue.

### Two-sample Mendelian randomization for inferring causal relationships among different conditions/diseases

Mendelian randomization (MR) uses genetic variants as instrumental variables to determine whether an observational association between a risk factor and an outcome is consistent with a causal effect [24]. Based on GWAS summary statistics, Mendelian randomization (MR) analyses were performed to detect potential causality between the 14 diseases. For instrumental variable (IV) selection, independent significant (IVs) (*P*<5E-08) and LD r^2^<0.01 within 10Mb based on the 1000Genomes Project phase 3 Europeans were kept [25]. Besides, we incorporated the F score to prevent weak instrument IVs (SNPs with F<10 were excluded) [26]. The inverse variance weighted method was applied to estimate the causal association between various diseases (IVW *P*<0.05), with a supplementary analysis of the weighted median, weight mode, and MR‒Egger. To address multiple hypothesis testing, we estimated the false discovery rate (FDR) adjusted p values, in the main IVW MR analyses, using the sequential p value approach proposed by Benjamini & Hochberg [27]. For the trait pairs with heterogeneity, we used IVW analysis with different models. Cochran *Q* statistics confirm the robustness of the MR estimates [28]. All analyses were performed by using the R package “TwoSampleMR” (version 0.5.6).

## Results

### Linkage disequilibrium score regression analysis (LDSC)

**Fig 1:**
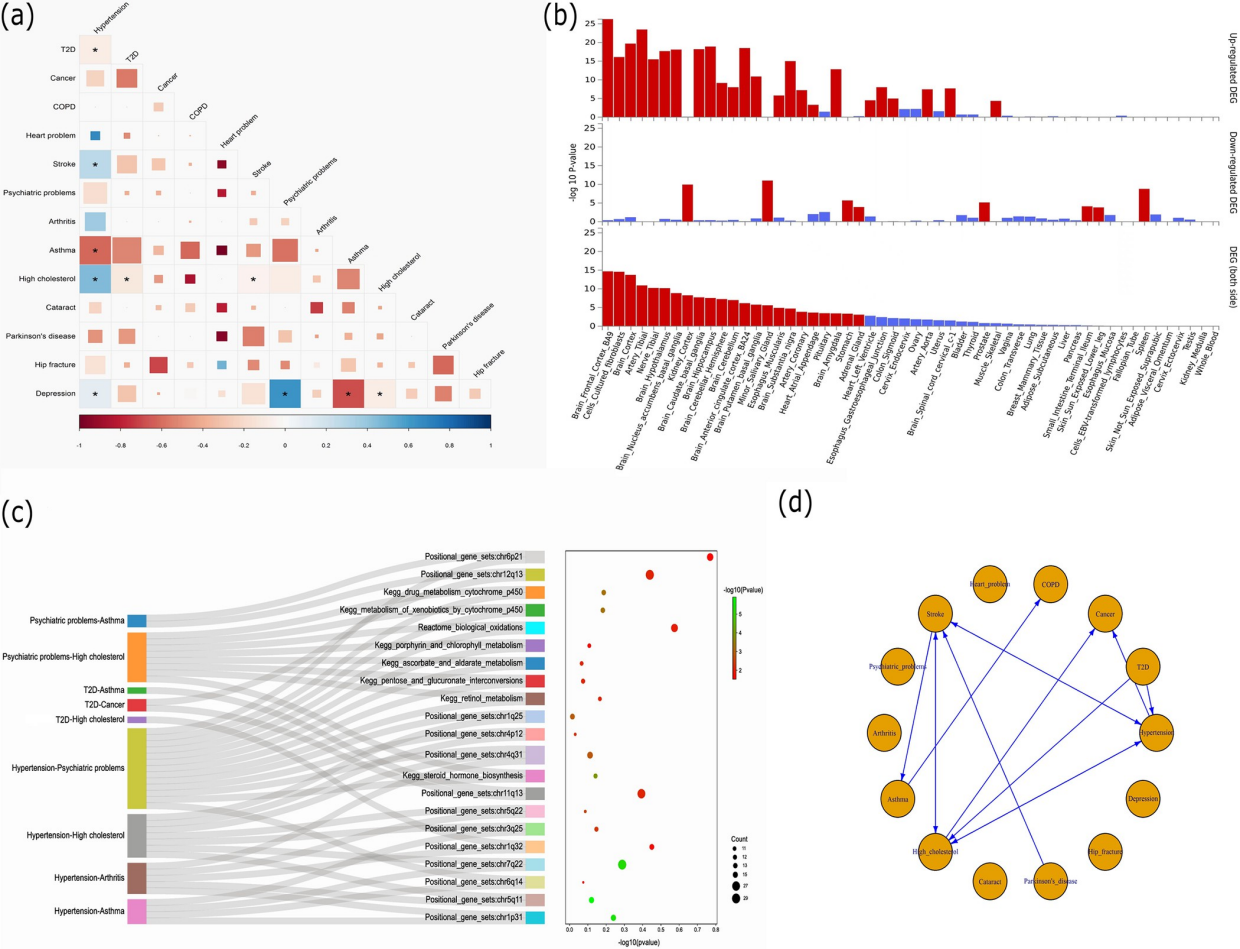
Genetic correlation analysis between 14 phenotypes. **(A)** LDSC results. Square color indicates r(g), blue indicates a positive correlation, and red indicates a negative correlation. Square size indicates Bonferroni-adjusted p value, * indicates *P*< 5.49E-04. Psychiatric problems: Mental health problems ever diagnosed by a professional: Anxiety, nerves or generalized anxiety disorder. **(B)** Differential expression levels of identified pleiotropic genes between 11 pairs of phenotypes in GTEx v8 54 tissue types. The -log10 (P value) in the graph refers to the probability of hypergeometric testing. Red bars denote tissues that remained significantly prominent after Bonferroni correction (P<0.05). **(C)** The shared enrichment pathways and/or GO terms. In the Sankey bubble diagram, the first column on the left side indicates each phenotypic pairwise combination. The second column represents the gene set enrichment analysis of the genes associated with the combination, in which different phenotypic combinations identified the same enrichment signal. The bubble diagram on the right side shows the genes enriched in the corresponding gene set. Positional gene sets are sets of genes that are categorized based on their different locations on the chromosome. Psychiatric problems: Mental health problems ever diagnosed by a professional: Anxiety, nerves or generalized anxiety disorder. **(D)** Network plot obtained from two-sample Mendelian randomization analysis for the 14 diseases/conditions (IVW *P*<0.05). The starting point of the connecting line represents exposure, and the end point of the arrow indicates the outcome. COPD: Chronic obstructive pulmonary disease. Psychiatric problems: Mental health problems ever diagnosed by a professional: Anxiety, nerves or generalized anxiety disorder.

LDSC estimated the genetic correlations among the 14 phenotypes (**S1 Table in S2 File**). Twenty pairs of conditions/diseases showed significant genetic correlations (*P* <0.05). The negative and positive coefficients ranged from -0.20~-0.08 and 0.16~0.75, respectively (**Table 2**). Among the 20 studied pairs, genetic correlations for 10 pairs were significant after Bonferroni adjustment (*P*<0.05/91 = 5.49E-04) (**Fig 1A**). We found that hypertension was significant genetic correlations with 5 diseases/conditions. It was positively genetically correlated with other 4 diseases/conditions, including T2D (rg = 0.23, *P* = 1.3E-08), stroke (rg = 0.49, *P* = 1.2E-21), high cholesterol (rg = 0.62, *P* = 3.2E-39) and depression (rg = 0.37, *P* = 1.3E-07). Besides, hypertension had a negative correlation with asthma (rg = -0.15, *P* = 2.0E-04). High cholesterol was positively genetically correlated with T2D (rg = 0.21, *P* = 4.7E-06), stroke (rg = 0.26, *P* = 2.05E-08) and depression (rg = 0.23, *P* = 1.0E-04). Depression was positively genetically correlated with psychiatric problems (mainly referring to anxiety and nerves) (rg = 0.75, *P* = 1.5E-16) and negatively correlated with asthma (rg = -0.20, *P* = 1.0E-04), respectively (**Table 2 and Fig 1A**).

**Table 2:** Genetic correlations among 14 conditions/diseases.

Trait1	Trait2	r(g)	se	z	p	h_1_^2^	h_2_^2^
Hypertension	T2D^a^	0.23	0.04	5.7	1.3E-08	0.038	0.058
Hypertension	Stroke	0.49	0.05	9.6	1.2E-21	0.038	0.011
Hypertension	Psychiatric problems	0.16	0.07	2.4	1.7E-02	0.038	0.025
Hypertension	Arthritis	0.56	0.28	2.0	4.4E-02	0.043	0.002
Hypertension	Asthma	-0.15	0.04	-3.8	1.1E-04	0.038	0.036
Hypertension	High cholesterol	0.62	0.05	13.1	3.2E-39	0.038	0.031
Hypertension	Depression	0.37	0.07	5.3	1.3E-07	0.038	0.024
T2D^a^	Cancer	-0.11	0.06	-2.0	4.6E-02	0.053	0.029
T2D^a^	Asthma	-0.08	0.03	-3.0	2.4E-03	0.05	0.042
T2D^a^	High cholesterol	0.21	0.05	4.6	4.7E-06	0.053	0.032
Stroke	High cholesterol	0.26	0.05	5.6	2.0E-08	0.011	0.030
Stroke	Parkinson’s disease	-0.10	0.05	-2.1	3.6E-02	0.012	0.012
Stroke	Depression	0.18	0.08	2.3	1.9E-02	0.009	0.026
Psychiatric problems	Asthma	-0.13	0.05	-2.6	9.7E-03	0.025	0.039
Psychiatric problems	High cholesterol	0.23	0.07	3.3	1.2E-03	0.026	0.031
Psychiatric problems	Depression	0.75	0.09	8.3	1.5E-16	0.026	0.026
Asthma	High cholesterol	-0.08	0.04	-2.2	2.7E-02	0.036	0.031
Asthma	Depression	-0.20	0.05	-3.4	1.0E-04	0.039	0.027
High cholesterol	Depression	0.23	0.06	3.8	1.0E-04	0.030	0.024
Parkinson’s disease	Hip fracture	-0.15	0.07	-2.0	4.8E-02	0.012	0.003

Note: T2D^a^: type 2 diabetes. Psychiatric problems: Mental health problems ever diagnosed by a professional: Anxiety, nerves or generalized anxiety disorder. r(g) indicates the estimate of genetic correlation and (±) indicates the direction of genetic correlation, se represents the standard error, z denotes z-score for genetic correlation h^2^ is the SNP-based heritability estimated by LDSC. Bolded P-values indicate trait pairs with significant genetic correlations in the 14 phenotypes (*P*<0.05), h_1_^2^ and h_2_^2^ represent the heritability for the first trait and the second trait, respectively.

### Functional annotation for pleiotropic SNPs and corresponding genes

Furthermore, PLACO was used to investigate pleiotropic SNPs for the significant pairs in the LDSC analysis (i.e., 20 condition pairs in **Table 2**). The results showed that 11/20 pairs of correlated traits had pleiotropic SNPs (**S2 Table in S2 File**). We further applied FUMA for functional annotation of the relevant SNPs. For the 11 pairs, a total of 8,927 lead SNPs were selected (**S3 Table in S2 File and S1 Fig in S1 File**). For the pleiotropic genes of 11 pairs, we performed tissue differential expression analysis in GTEx v8 54 tissue types and showed that the pleiotropic genes were mainly enriched in the brain, nerves, kidney, heart, and blood vessels, particularly for these up-regulated pleiotropic genes (**Fig 1B**), indicating that these genes probably play important biological functions. We also performed enrichment analysis for each significant pair (**S4 Table in S2 File**) and identified a total of 21 common sets of genes involved in at least two pairs. Seven significant KEGG pathways were shared pathways, such as drug metabolism cytochrome p450 and porphyrin and chlorophyll metabolism (**Fig 1C**).

For a more detailed explanation of the pleiotropic SNPs and corresponding genes, we further divided these associated traits into five subgroups.

#### Hypertension-associated subgroup

The hypertension-associated group consisted of hypertension-psychiatric problems (anxiety and nerves), hypertension-arthritis, hypertension-asthma, and hypertension-high cholesterol. The pleiotropic SNPs identified by PLACO analysis were mapped to 1,030 pleiotropic genes (**S5 Table in S2 File**). The gene ontology (GO) gene sets showed that remarkably strong enrichment signals in the glucuronation and metabolism for the biological process (BP) terms. (i.e., GO_FLAVONOID_GLUCURONIDATION, *P*adj = 1.36E-04; GO_URONIC_ACID_METABOLIC_PROCESS, *P*adj = 5.58E-03;); For the KEGG, which them mainly were abundant in glucuronate interconversions and metabolism, (i.e., KEGG_PENTOSE_AND_GLUCURONIDATE_INTERCONVERSION, *P*adj = 5.99E-08; KEGG_STARCH_AND_SUCROSE_METABOLISM, *P*adj = 1.25E-04.) (**S6 Table in S2 File and S2 Fig in S1 File**). We found that the identified pleiotropic genes were significantly enriched in the brain, kidney, heart, and nerve tissue (**S3A Fig in S1 File**).

**T2D-associated subgroup.** A total of 1,582 unique pleiotropic genes were identified in the T2D-related cluster, which included T2D-cancer, T2D-asthma, and T2D-high cholesterol (**S7 Table in S2 File**). We found that the 1,582 genes showed strong enrichment signals in gene sets related to oncogenic transcription factors and the biocarta pathway (**S8 Table in S2 File**). Pleiotropic genes were mainly concentrated in brain tissue, down-regulated in the kidney and spleen, and up-regulated in vascular tissue and muscle (**S3B Fig in S1 File**).

#### Psychiatric problems-related group

The psychiatric problems-associated group mainly refers to phenotypes associated with anxiety and nerves, including asthma and high cholesterol. A total of 1,455 unique pleiotropic genes were identified by positional mapping and eQTL mapping (**S9 Table in S2 File**). For the biological process (BP) terms of GO, which them mainly were associated with adhesion pathways (i.e., GO_BIOLOGICAL_ADHESION, *P*adj = 1.36E-04; GO_CELL_CELL_ADHESION, *P*adj = 4.22E-06). For the molecular function (MF) terms of GO, these gene sets were primarily involved in synapses and neurotransmission pathways (i.e., GO_SYNAPSE, *P*adj = 2.32E-03; GO_NEURON_PART, *P*adj = 1.72E-02 (**S10 Table in S2 File and S4 Fig in S1 File**), which accumulate in brain and nerve tissues. **(S3C Fig in S1 File)**.

#### High cholesterol-depression subgroup

A total of 303 unique genes were identified by positional mapping and eQTL mapping between high cholesterol and depression (**S11 Table in S2 File**). These genes were enriched in diseases or mutants of signal transduction and associated with apoptosis (**S12 Table in S2 File and S5 Fig in S1 File**). However, they did not show significant differences in 30 tissues **(S3D Fig in S1 File)**.

#### Parkinson’s disease-hip fracture subgroup

There were 985 unique putative pleiotropic genes detected between Parkinson’s disease and hip fracture via two mapping methods (**S13 Table in S2 File**). The Gene Ontology (GO) gene sets showed that 985 unique genes had significant enrichment signals in neurons and cells (e.g., GO_REGULATION_OF_CELL_PROJECTION_ORGANIZATION, *P*adj = 1.15E-04; GO_CELL_PROJECTION_ORGANIZATION, *P*adj = 2.13E-04) **(S14 Table in S2 File and S6 Fig in S1 File)**. Interestingly, differential expression of these unique genes was significantly enriched in the prostate **(S3E Fig in S1 File).**

### Causal associations among 14 conditions/diseases inferred by Mendelian randomization analysis

**Fig 2:**
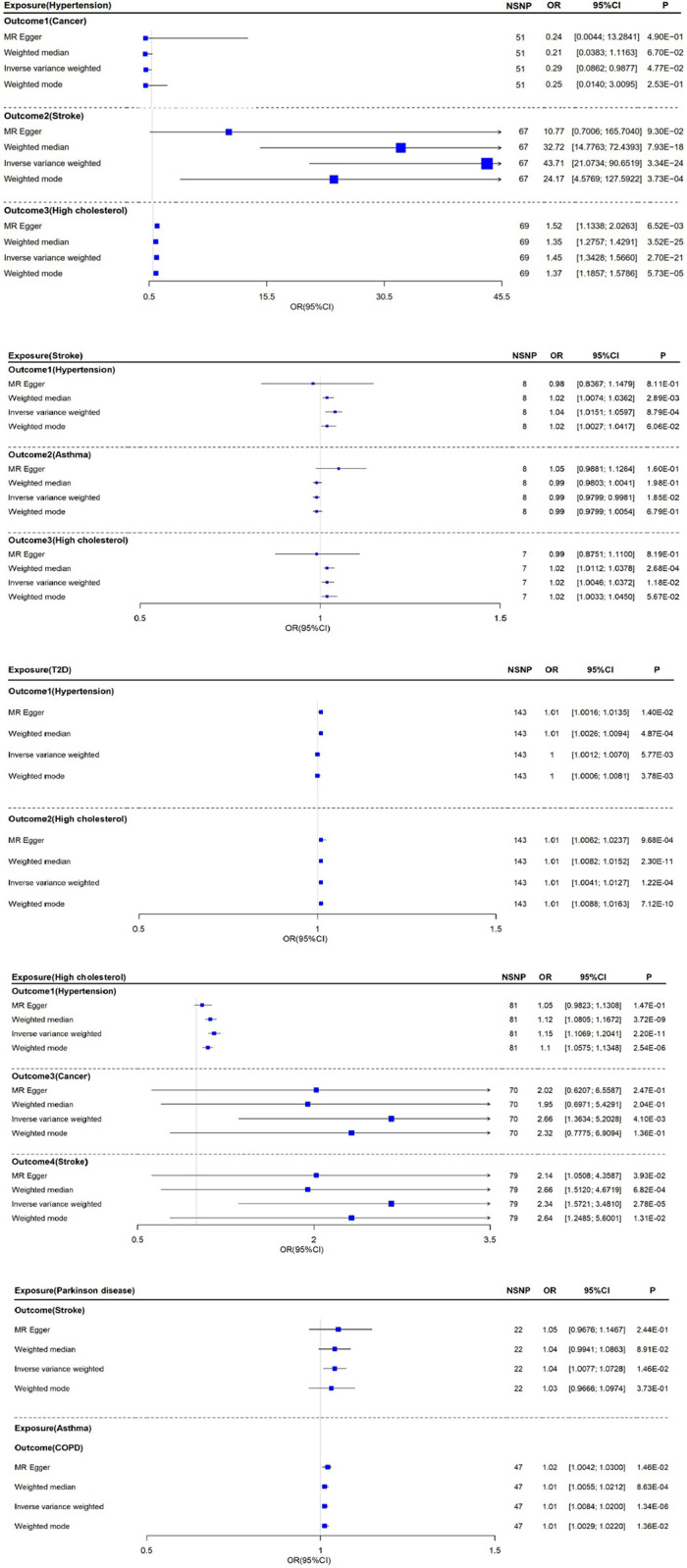
The forest figure shows the causal effects of different conditions/diseases as outcome variables at exposure (IVW P<0.05). It contained the ratio of exposure to outcome (OR) with a 95% confidence interval (95% Cl). Blue squares indicate ORs, and the area of the square indicates the size of the OR value; the larger the OR value is, the larger the area of the square.

Finally, our study systematically analyzed the bidirectional causal relationships among 14 conditions/diseases. Of all 182 examined associations, 82 pairs had instrumental variables with F-values larger than 10 (**S15 Table in S2 File**). Among these 82 pairs, we found 13 pairs with significant causal effects (IVW *P*<0.05). Notably, IVW analysis demonstrated that the three phenotypes (hypertension, stroke, and high cholesterol) not only displayed significant bidirectional causal associations among them but also showed unidirectional causal associations from these three phenotypes to others, which indicated that they increased the risk of other diseases **(Figs 1D and 2)**. After adjustments of *P*-values, eight pairs of traits still had significantly causal relationships: hypertension→stroke (*P*adj = 2.73E-22), hypertension→high cholesterol (*P*adj = 1.11E-19), T2D→high cholesterol (*P*adj = 1.67E-03), stroke→hypertension (*P*adj = 1.03E-02), high cholesterol→hypertension (*P*adj = 6.01E-10), high cholesterol→cancer (*P*adj = 4.20E-02), high cholesterol→stroke (*P*adj = 4.56E-04), asthma→COPD (*P*adj = 2.75E-05). However, there was no significant causal relationship between T2D and hypertension (*P*adj = 5.26E-02) and when stroke was the exposure and high cholesterol was the outcome, the *P*adj = 9.68E-02, suggesting that the causality between high cholesterol and stroke may be unidirectional (**S16 Table in S2 File**). In addition, MR‒Egger regression analyses showed no potential pleiotropy (all *P*_intercept_ > 0.05), and Cochran’s *Q* test indicated that heterogeneity was present in more than half (61.54%) of 13 pairs. For disease pairs with heterogeneity (*P*heter<0.05), the P-value of IVW with random effects was selected; for those without heterogeneity, the P-value of IVW with fixed effects was selected (*P*heter>0.05) (**S17 Table in S2 File**). Scatter plots of the MR results are provided in the supplementary materials (**S7 Fig in S1 File**).

Since the phenotypic correlations were probably caused by shared genetic effects and/or significant causal effects, we summarized the underlying mechanisms for the 24 studied pairs (**Table 3**). The phenotypic correlations for six pairs (hypertension & T2D, hypertension & stroke, hypertension & high cholesterol, T2D & high cholesterol, stroke & high cholesterol, and stroke & Parkinson’s disease) were probably caused by both shared genetic effects and significant causal effects, but for other pairs, their correlations were probably caused by either shared genetic effects or significant causal effects. Hypertension, stroke, and high cholesterol have been frequently observed to be associated with other conditions/diseases.

**Table 3:** The significant results of LDSC and MR analyses for the significant phenotypic pairs.

Phenotype1	Phenotype2	LDSC	MR
Hypertension	T2D^a^	+	+ (2→1)
Hypertension	Cancer	−	+ (1→2)
Hypertension	Stroke	+	+ (1↔2)
Hypertension	Psychiatric problems	+	−
Hypertension	Arthritis	+	−
Hypertension	Asthma	+	−
Hypertension	High cholesterol	+	+ (1↔2)
Hypertension	Depression	+	−
T2D^a^	Cancer	+	−
T2D^a^	Asthma	+	−
T2D^a^	High cholesterol	+	+ (1→2)
Stroke	Asthma	−	+ (1→2)
Stroke	High cholesterol	+	+ (1↔2)
Stroke	Parkinson’s disease	+	+ (2→1)
Stroke	Depression	+	−
High cholesterol	Psychiatric problems	+	−
High cholesterol	Cancer	−	+ (1→2)
High cholesterol	Asthma	+	−
High cholesterol	Depression	+	−
Psychiatric problems	Asthma	+	−
Psychiatric problems	Depression	+	−
Asthma	COPD^b^	−	+ (1→2)
Asthma	Depression	+	−
Parkinson’s disease	Hip fracture	+	−

Note: "+" represents the trait pairs with a significant relationship by LDSC and/or MR analysis (*p<0.05), while "-" indicates no significant association (*p*>0.05); The direction of the arrow reflected causality from exposure to outcome; T2D^a^: type 2 diabetes; COPD^b^: chronic obstructive pulmonary disease. Psychiatric problems: Mental health problems ever diagnosed by a professional: Anxiety, nerves or generalized anxiety disorder.

## Discussion

This study performed systemic analyses to detect the shared genetic and causal effects of 14 common conditions/diseases in multimorbidity patterns. LDSC analysis showed that the 20 studied pairs were genetically correlated, indicating that genetic effects were shared by the 14 conditions/diseases. The pleiotropic SNPs identified by PLACO and the corresponding mapping genes overlapped among these studied phenotypic pairs and were enriched in some pathways and/or GO terms with important biological functions. Compared to others, hypertension, stroke, and high cholesterol were observed to be causally/genetically associated with other phenotypes at a high frequency, and they most likely serve as the driving factors for other conditions/diseases.

Previous GWASs have shown that numerous genomic loci are significantly associated with a large diversity of traits [29] Extensive pleiotropic effects were identified as a possible explanation for comorbidity. This finding pointed to potential common genetic mechanisms between diseases. Most importantly, we found that hypertension, stroke, and high cholesterol were associated with more than one phenotype, emphasizing their prominence in the comorbidity pattern [30]. Among ~380 chronic disease combinations identified in older adults, hypertension was present in almost every combination of prevalent diseases [31].

Functional annotation analysis showed that putative pleiotropic genes of 11 pairs of traits were involved in biological processes such as biotransformation (e.g., glucuronate pathway, drug metabolism). The glucuronate pathway is indispensable for the physiological function of the body and has the functions of detoxification and excretion, and the metabolism of many drugs depends on it [32]. Several chronic inflammatory diseases (such as metabolic syndrome, T2D, and cardiovascular disease) could affect glucose metabolism [33]. We identified 985 pleiotropic genes in Parkinson’s disease and hip fracture that were involved in the development of neurons. Previous studies have shown that neuronal damage in Parkinson’s disease leads to symptoms such as tremors and muscle stiffness, which contribute to an increased number of falls and are also associated with fracture risk [34]. The above findings suggest that neuronal regulation plays an important role in the pathophysiology of both Parkinson’s disease and hip fracture.

The pleiotropic genes were mainly enriched in the brain, nerves, kidney, heart, and blood vessels. Previous evidence supports that vascular dysfunction is a common feature of several chronic diseases [35]. For example, hypertension, T2D, and kidney diseases were proven to be associated with cerebrovascular damage [36]. Chronic inflammatory diseases could induce higher chemokine production and activate intrarenal macrophages and dendritic cells in rats [37]. This suggested that the kidneys may be involved. In addition, we found that some disease pairs shared common biological functions. Seven KEGG pathways were associated with different trait pairs. The pathways involved in drug metabolism by cytochrome p450 and the metabolism of xenobiotics by cytochrome p450 were related to the balance between the oxidative stress system and antioxidant capacity and might play a vital role in hypertension-related conditions/diseases [38]. In addition, another study found that both hypertension and high cholesterol in animal models lead to increased systemic and vascular oxidative stress, resulting in reduced vasodilatation [39]. The oxidative system is closely associated with hypertension and T2D [40]. Based on the above evidence, we speculated that the dysregulation of the oxidative stress system may represent the major characteristics of the pathological process of hypertension-related diseases. The metabolism of porphyrin and chlorophyll and the metabolism of ascorbic acid and erythrosine in two trait pairs (hypertension—psychiatric problems and psychiatric problems—high cholesterol) may be newly discovered pathways because they have not been previously reported (psychiatric problems mainly refer to anxiety and nerves).

The LDSC analysis showed that hypertension was genetically related to T2D. The Mendelian randomization method demonstrated that T2D had a unidirectional causal effect on hypertension. Previous MR analyses have shown similar results. Zhu et al. used community-based disease GWAS data (p = 0.44 for SBP→T2D and p = 0.20 for DBP→T2D) [41], which showed no causal relations from hypertension to T2D, which was in line with our results with no adjustment. However, after correction, there was no causal effect of T2D on hypertension, thus, caution should be exercised when considering the causality of the two phenotypes. A cross-sectional study showed that clinically significant depressive symptoms were associated with elevated blood pressure, but they could not determine any causal relationship between hypertension and depression [42]. Our MR analysis showed that there was no causal association between them. In comparison, LDSC showed a strong genetic correlation between them (r(g) = 0.37, *P* = 1.30E-07). Evidence suggests that endothelial dysfunction plays an important role in the pathobiology of depression [43]. The vascular endothelium regulates vascular homeostasis and is a key factor in vascular health [44]. Therefore, we speculate that hypertension could lead to decreased vascular function and endothelial damage, which increases the risk of developing depression.

Hypertension and high cholesterol are common clinical comorbidities that have been previously described [45]. Hernández et al. analyzed the chronic combination pattern of 6,101 older adults aged 50 years and older and found that hypertension and high cholesterol were the most common coexisting conditions/diseases [46]. The 267 pleiotropic genes shared between them were mainly enriched in the synthesis and degradation of fibrinogen complexes, which have been reported to play an important role in high cholesterol patients with hypertension [47]. There also existed bidirectional causality between hypertension and stroke. Both the LDSC and MR results suggest that there are shared genetic determinants between the two phenotypes. A meta-analysis revealed that for every 10 mmHg increase in systolic blood pressure, the risk of stroke increased by 22%, and mortality increased by 56% [48]. This may be attributed to the possibility that hypertension accelerates the atherosclerotic process, thus increasing the probability of stroke. We confirmed the association from a genetic perspective.

We noticed that no pleiotropic SNPs/genes were found between hypertension and stroke or between stroke and high cholesterol. The possible reason is that we conducted SNP filtering under stricter thresholds (P<5E-08), which helped increase the efficacy of the test but also led to the removal of real pleiotropic SNPs. Moreover, the pleiotropy of complex traits/diseases is an essential basis for genetic correlations between diseases [49]. Hypertension, T2D, and high cholesterol seem to occupy an influential position in comorbidity patterns, which are presumably the dominant diseases to study the patterns of other conditions/diseases. Due to the uncertainty that all confounding factors have been identified, observational trials studying associations between diseases are susceptible to interference by many external factors, such as medications [50]. For example, certain diseases are bad outcomes for the treatment of specific diseases (e.g., clozapine and olanzapine, used for treating psychiatric disorders, can lead to insulin sensitivity and lipid metabolism changes, which increase the risk of diabetes and cardiovascular disease) [50]. However, this co-occurrence is not a genetic association of the diseases themselves but rather an iatrogenic condition resulting from psychiatric medications. Therefore, even if a strong statistical association between exposure and outcome is observed, causal conclusions should be drawn with caution. The present study assessed the relationship between diseases by using an associated genetic variation, which is usually irrespective of environmental or lifestyle factors and could strengthen causal inference. An in-depth analysis of correlations between the disease/conditions from the perspective of genetic variation, identification of pleiotropic genes and biological pathways, and causal effects was conducted to provide genetic insights for observational studies.

However, there are two limitations to our study. First, the summary data in our study only represent European populations; thus, caution should be taken when generalizing the findings to other ethnic groups (e.g., East Asians). Second, although we used multiple MR approaches to mitigate confounding due to pleiotropy, residual bias cannot be entirely ruled out, as it is an established limitation of the MR methodology [51].

## Conclusion

The high phenotypic correlations for the 14 conditions/diseases in the multimorbidity patterns were caused by shared genetic effects and/or significant causal effects. Compared to others, hypertension, stroke, and high cholesterol were most frequently observed to be causally/genetically associated with other phenotypes, and they most likely serve as the driving factors for other conditions/diseases. These findings may provide basic data for the prevention, diagnosis, and treatment of these conditions/diseases.

## Supporting information

S1 Checklist Human participants research checklist. (DOCX)

S1 File (PDF)

S2 File (XLSX)
