# Supplementary material for: Genetic effects and causal association analyses of 14 common conditions/diseases in multimorbidity patterns
Source: PLoS One. 2024 May 16;19(5):e0300740. doi: 10.1371/journal.pone.0300740 (PMC11098521; doi:10.1371/journal.pone.0300740)
Supplement: S1 File — (PDF) [file pone.0300740.s002.pdf]

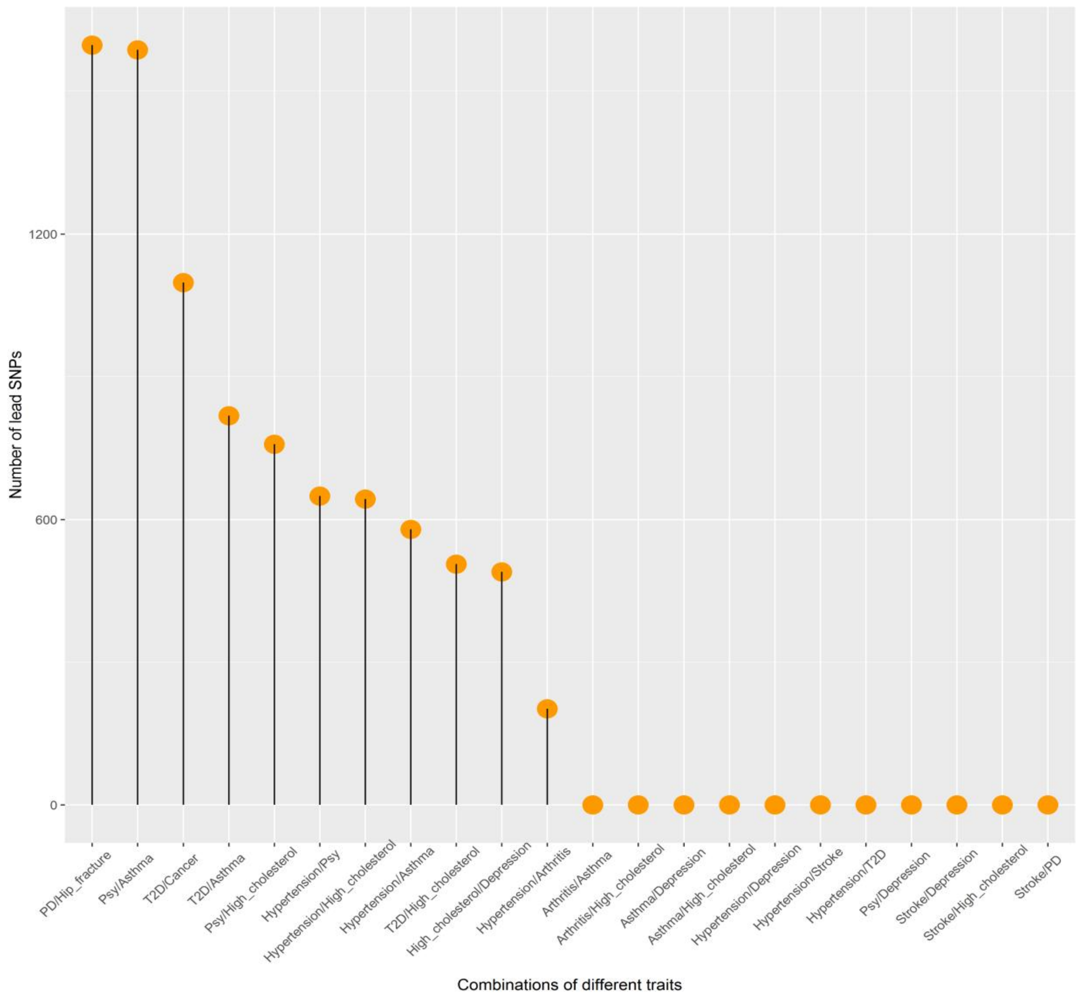

S1 Fig. Number of lead SNPs for 11 pairs of phenotypes identified by FUMA based on PLACO analysis. PD: Parkinson's disease. Psy: Psychiatric problems, which mainly refer to anxiety and nerves.

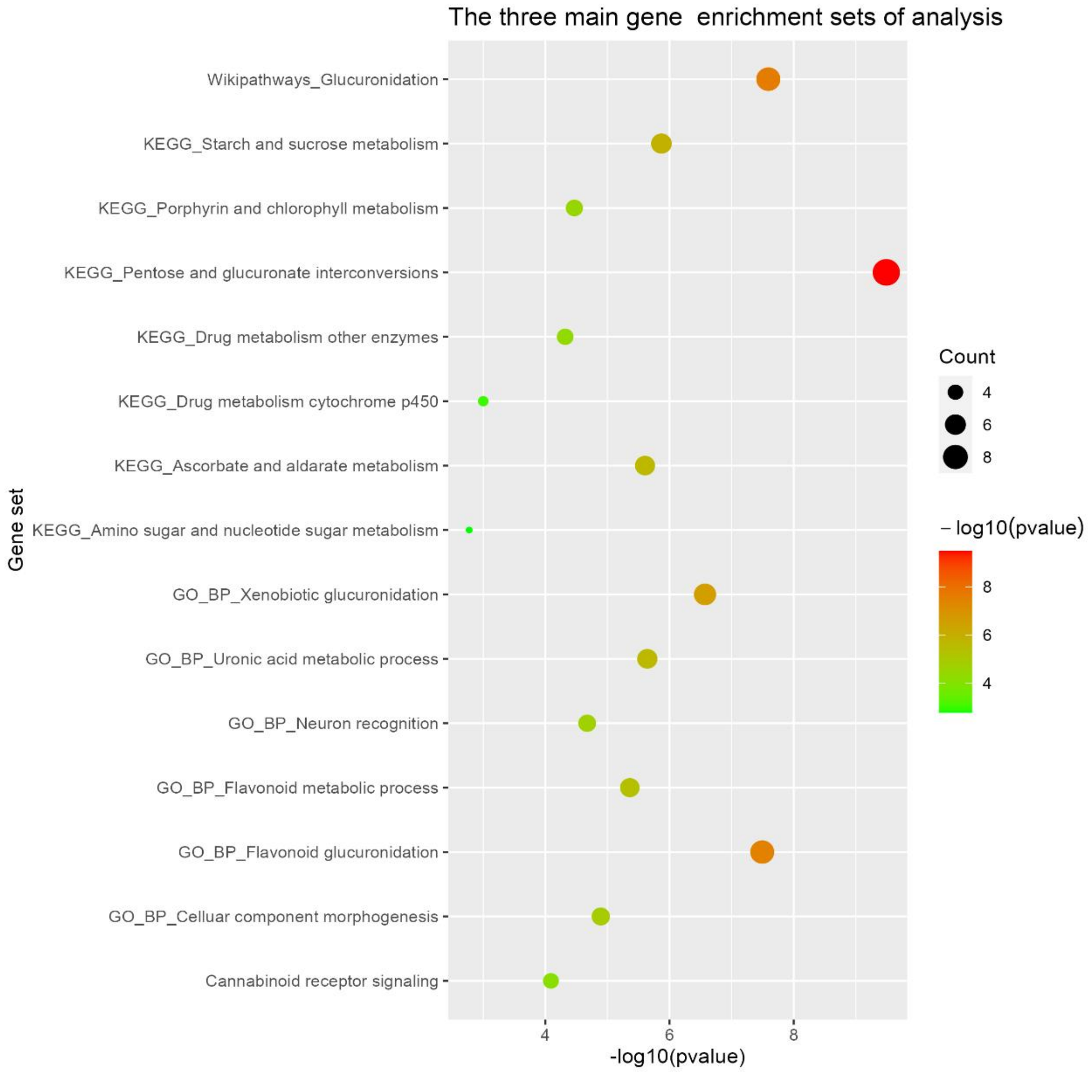

S2 Fig. In the bubble plot enrichment analysis of hypertension-associated group, the X-axis was the –log10 value transformed by the p-value, the larger the value, the higher the degree of gene enrichment of the pathway. Y-axis indicated the name of the enriched gene set pathway. The size of the dot indicated the number of Gene, the larger the dot, the more genes were enriched to that pathway. The color represented the level of P-value, the larger the -log10(Pvalue), the smaller the P-value, the more significant the pathway.

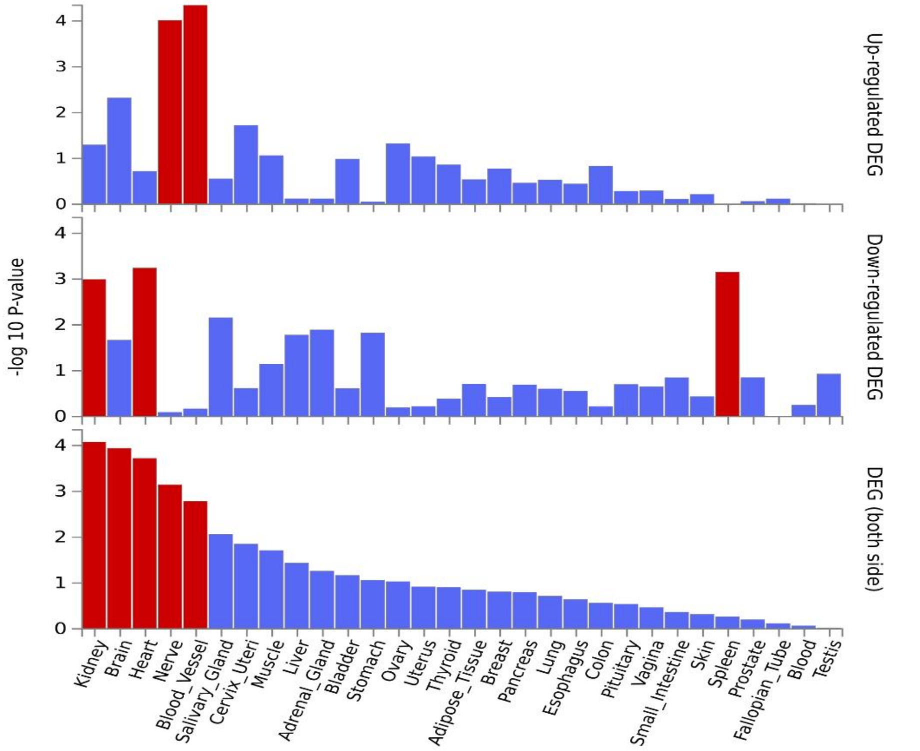
-log 10 P-value
Up-regulated DEG
Down-regulated DEG
DEG (both side)
0
1
2
3
4
Kidney
Brain
Heart
Nerve
Blood_Vessel
Salivary_Gland
Cervix_Uteri
Muscle
Liver
Adrenal_Gland
Bladder
Stomach
Ovary
Uterus
Thyroid
Adipose_Tissue
Breast
Pancreas
Lung
Esophagus
Colon
Pituitary
Vagina
Small_Intestine
Skin
Spleen
Prostate
Fallopian_Tube
Blood
Testis

(A)

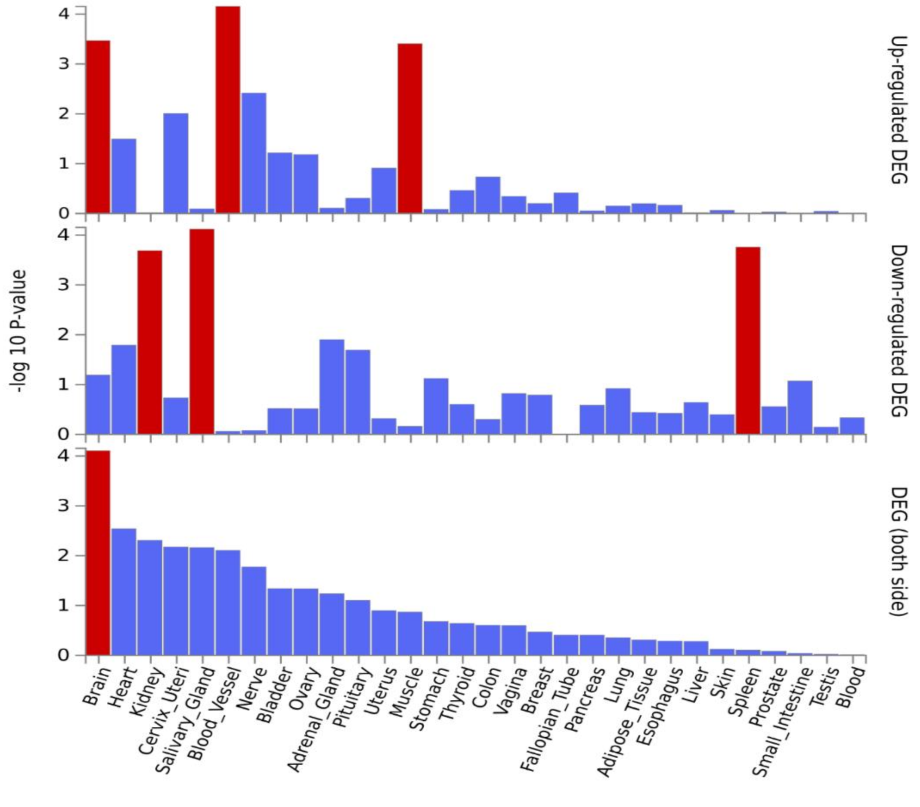
-log 10 P-value
Up-regulated DEG
Down-regulated DEG
DEG (both side)
0
1
2
3
4
Brain
Heart
Kidney
Cervix_Uteri
Salivary_Gland
Blood_Vessel
Nerve
Bladder
Ovary
Adrenal_Gland
Pituitary
Uterus
Muscle
Stomach
Thyroid
Colon
Vagina
Breast
Fallopian_Tube
Pancreas
Lung
Adipose_Tissue
Esophagus
Liver
Skin
Spleen
Prostate
Small_Intestine
Testis
Blood

(B)

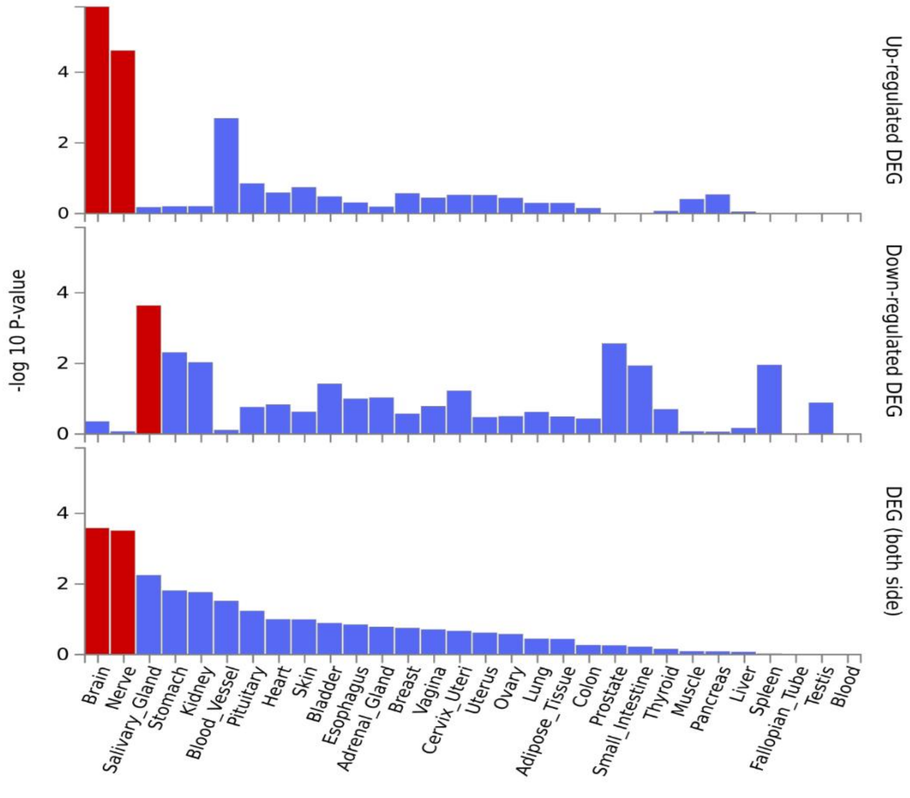
Up-regulated DEG
Down-regulated DEG
DEG (both side)
-log 10 P-value
Brain
Nerve
Salivary_Gland
Stomach
Kidney
Blood_Vessel
Pituitary
Heart
Skin
Bladder
Esophagus
Adrenal_Gland
Breast
Vagina
Cervix_Uteri
Uterus
Ovary
Lung
Adipose_Tissue
Colon
Prostate
Small_Intestine
Thyroid
Muscle
Pancreas
Liver
Spleen
Fallopian_Tube
Testis
Blood

(C)

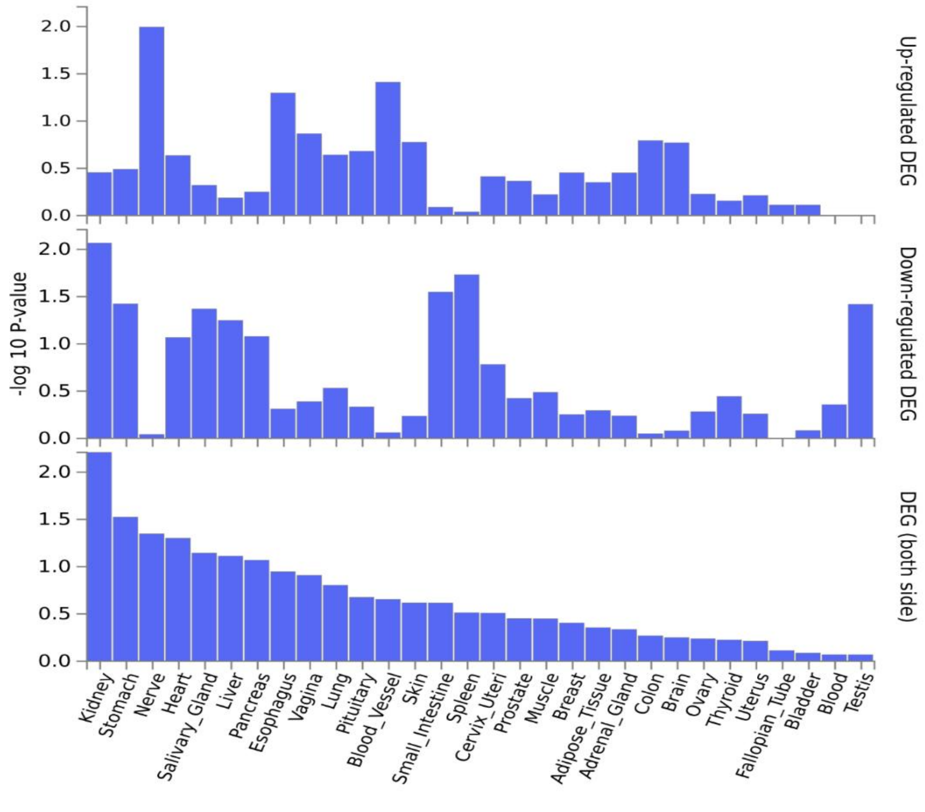
Up-regulated DEG
Down-regulated DEG
DEG (both side)
-log 10 P-value
Kidney
Stomach
Nerve
Heart
Salivary_Gland
Liver
Pancreas
Esophagus
Vagina
Lung
Pituitary
Blood_Vessel
Skin
Small_Intestine
Spleen
Cervix_Uteri
Prostate
Muscle
Breast
Adipose_Tissue
Adrenal_Gland
Colon
Brain
Ovary
Thyroid
Uterus
Fallopian_Tube
Bladder
Blood
Testis

(D)

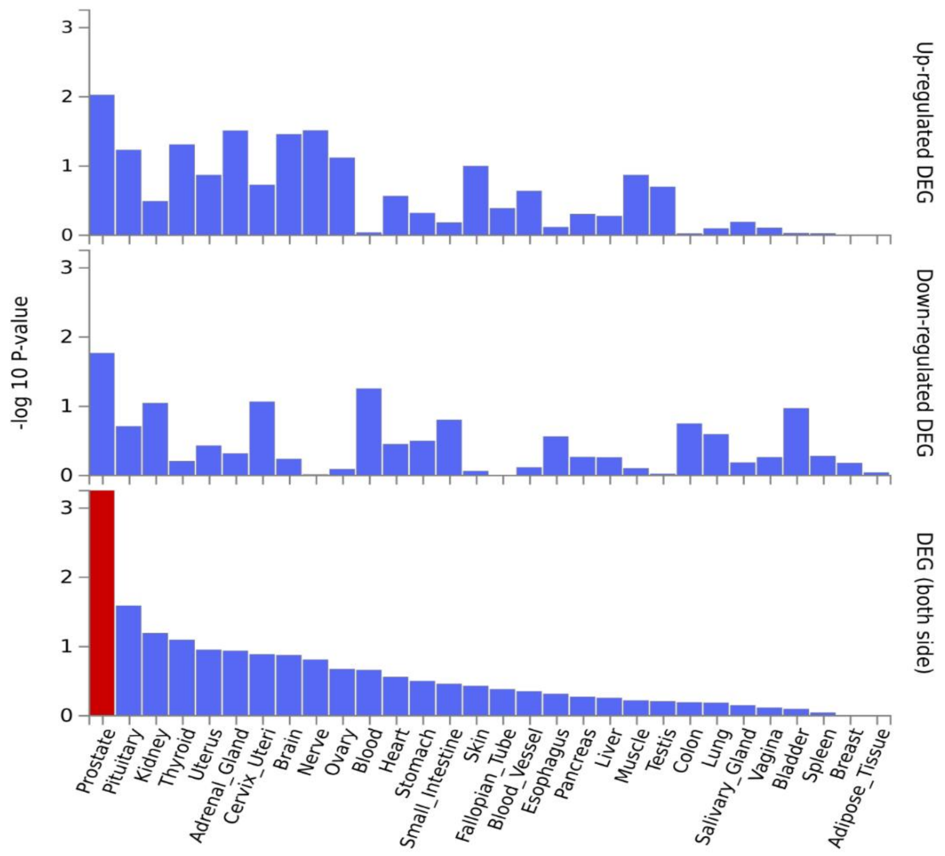

(E)

S3 Fig. Differential expression levels of identified unique pleiotropic genes between different group in 30 GTEx tissues. The -log10 (p-value) in the graph refers to the probability of hypergeometric testing. Red bars denote tissues that remain significantly prominent after correction using Bonferroni (P<0.05). In contrast, the blue bars are tissues that were corrected for the absence of differential expression. **(A)** Tissue differential expression levels of genes in groups associated with hypertension. **(B)** The differentially expressed in T2D-associated group. **(C)** Differential expression of pleiotropic genes in psychiatric-related groups. Psychiatric problems: Mental health problems ever diagnosed by a professional: Anxiety, nerves or generalized anxiety disorder. **(D)** Tissue differential expression levels of genes between high cholesterol and depression. **(E)** the expression-level of pleiotropic genes between Parkinson's disease and hip fracture in 30 tissues.

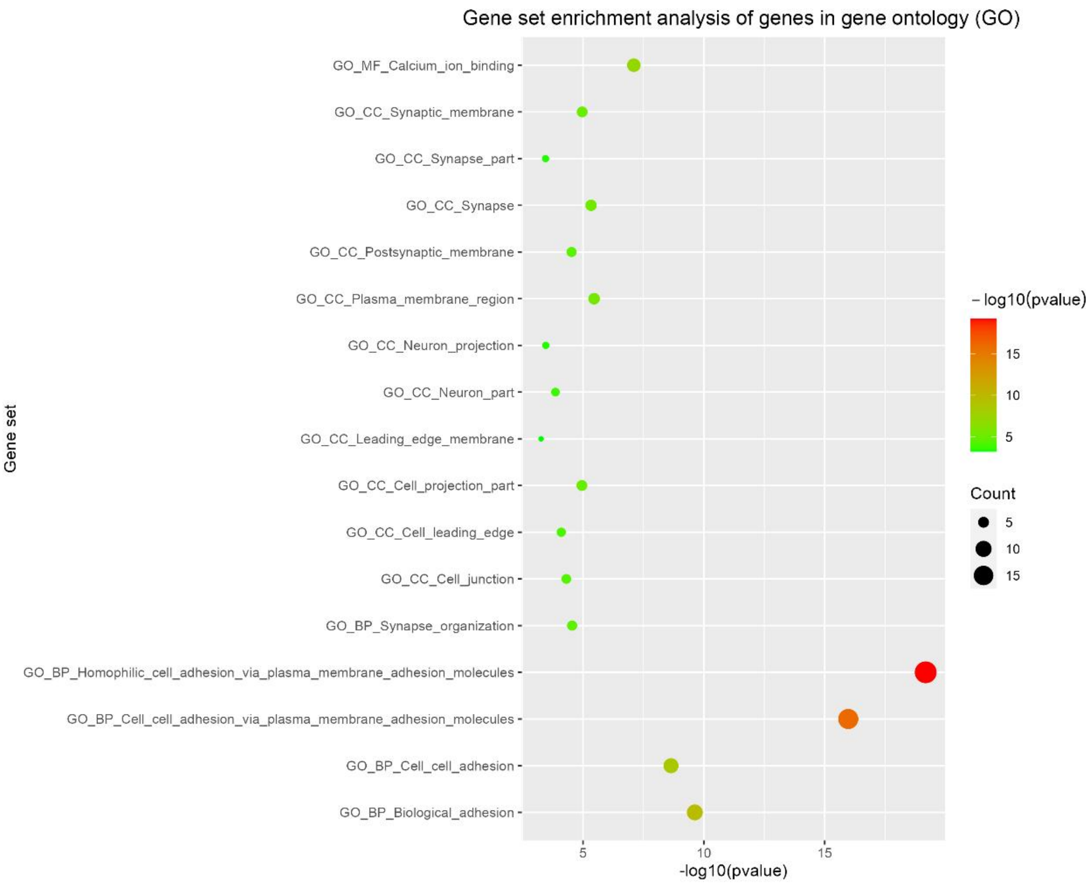

S4 Fig. In the bubble plot enrichment analysis associated with psychiatric problems-related group. Psychiatric problems: Mental health problems ever diagnosed by a professional: Anxiety, nerves or generalized anxiety disorder. The x-axis is the –log10 value transformed by the p-value, the larger the value, the higher the degree of gene enrichment of the pathway. y-axis indicates the name of the enriched gene set pathway. The size of the dot indicates the number of Gene, the larger the dot, the more genes are enriched to that pathway. The color represents the level of P-value, the larger the -log10(Pvalue), the smaller the P-value, the more significant the pathway.

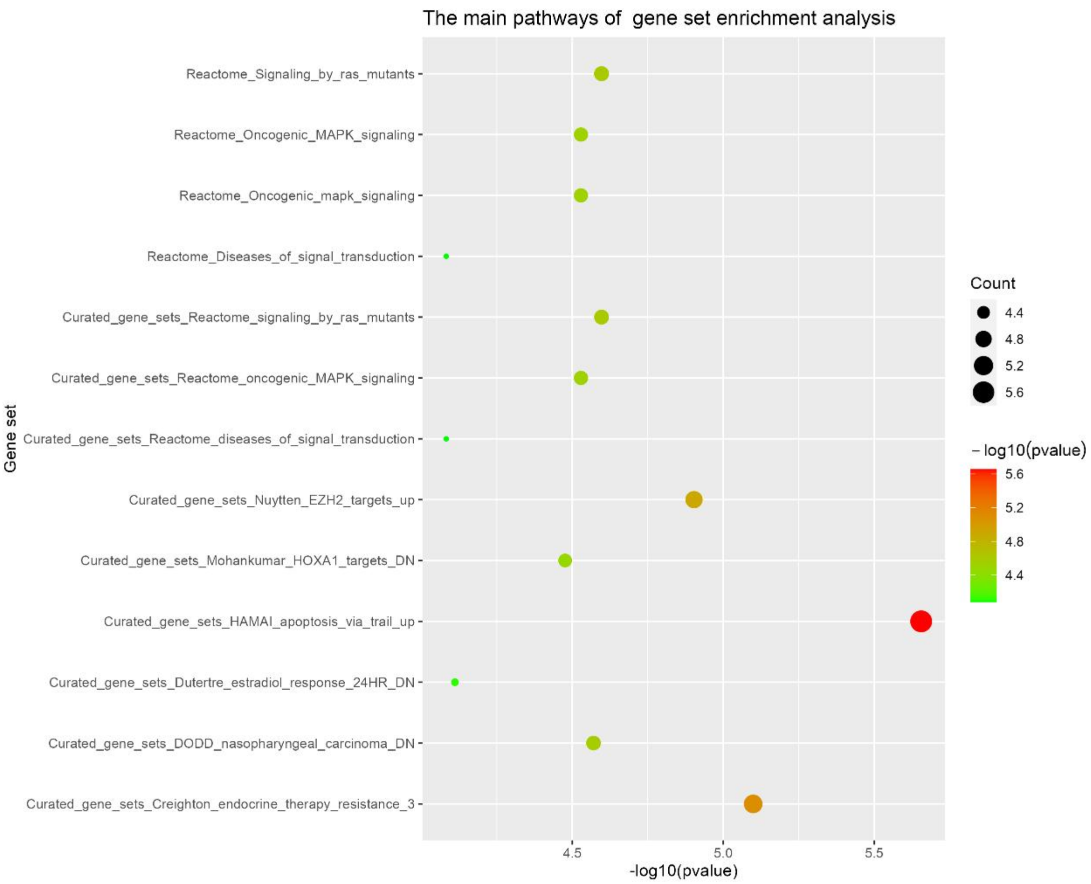

S5 Fig. Bubble plot enrichment analysis of high cholesterol and depression. The x-axis is the –log10 value transformed by the p-value, the larger the value, the higher the degree of gene enrichment of the pathway. y-axis indicates the name of the enriched gene set pathway. The size of the dot indicates the number of Gene, the larger the dot, the more genes are enriched to that pathway. The color represents the level of P-value, the larger the -log10(Pvalue), the smaller the P-value, the more significant the pathway.

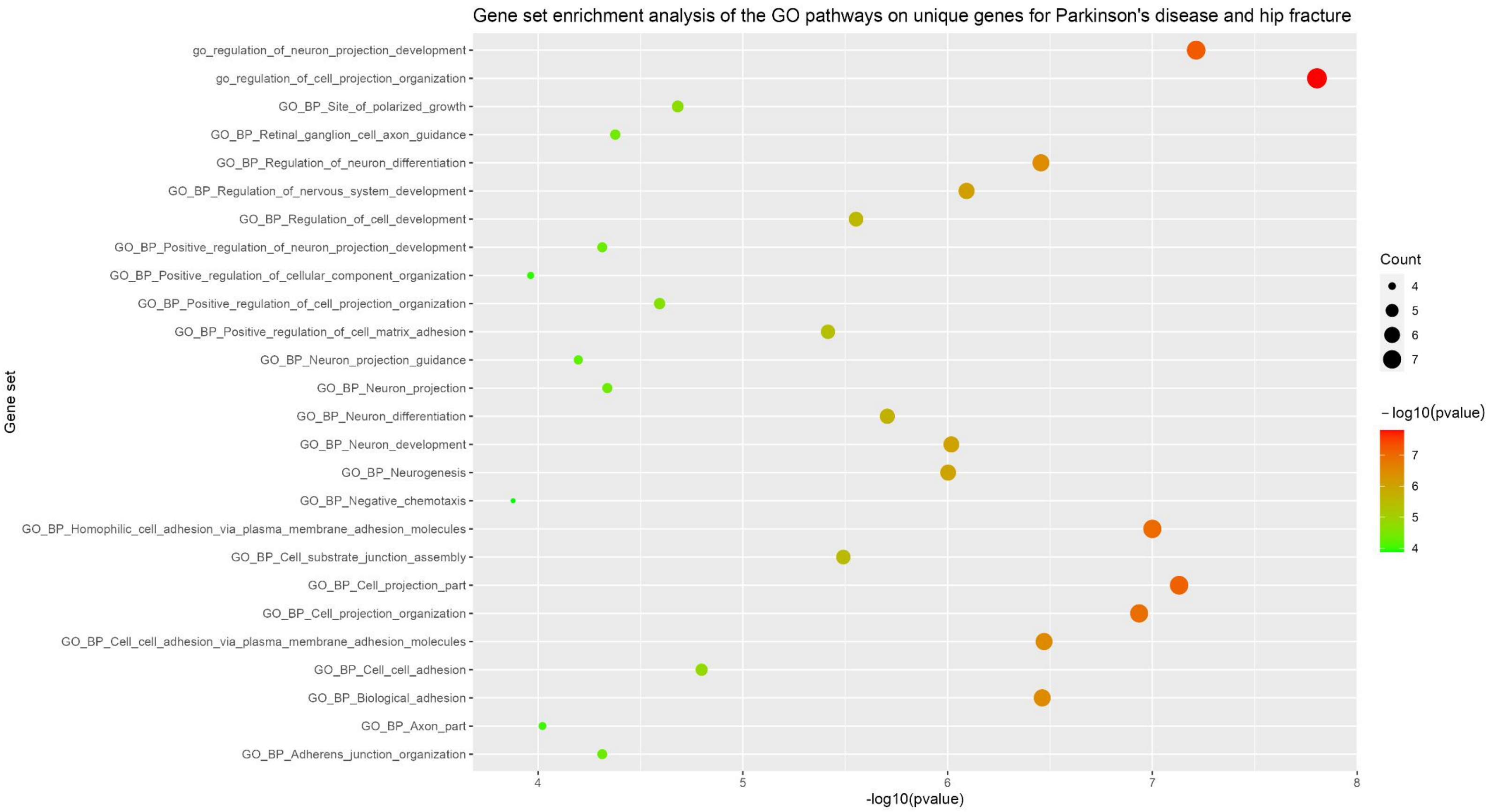

S6 Fig. Bubble plot enrichment analysis of Parkinson’s disease and hip fracture. The x-axis is the –log10 value transformed by the p-value, the larger the value, the higher the degree of gene enrichment of the pathway. y-axis indicates the name of the enriched gene set pathway. The size of the dot indicates the number of Gene, the larger the dot, the more genes are enriched to that pathway. The color represents the level of P-value, the larger the -log10(Pvalue), the smaller the P-value, the more significant the pathway.

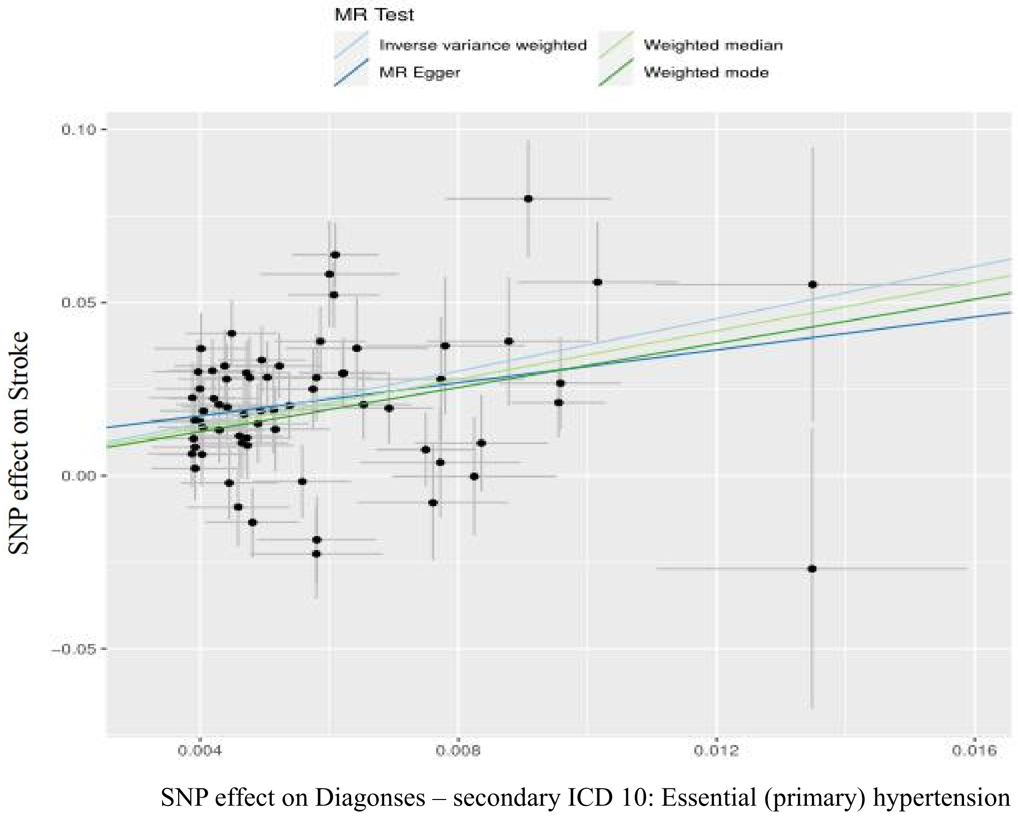
MR Test
Inverse variance weighted
MR Egger
Weighted median
Weighted mode
0.10
0.05
0.00
−0.05
0.004
0.008
0.012
0.016
SNP effect on Stroke
SNP effect on Diagonses – secondary ICD 10: Essential (primary) hypertension

(A)

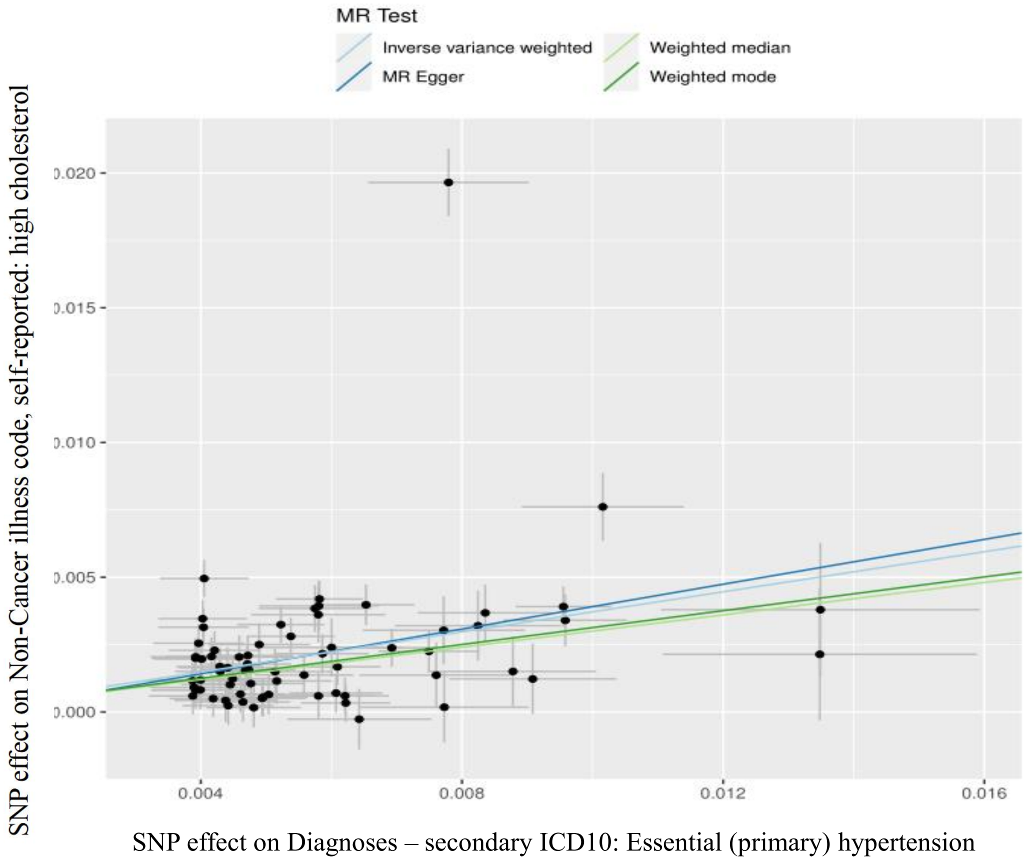
MR Test
Inverse variance weighted
MR Egger
Weighted median
Weighted mode
0.020
0.015
0.010
0.005
0.000
0.004
0.008
0.012
0.016
SNP effect on Non-Cancer illness code, self-reported: high cholesterol
SNP effect on Diagnoses – secondary ICD10: Essential (primary) hypertension

(B)

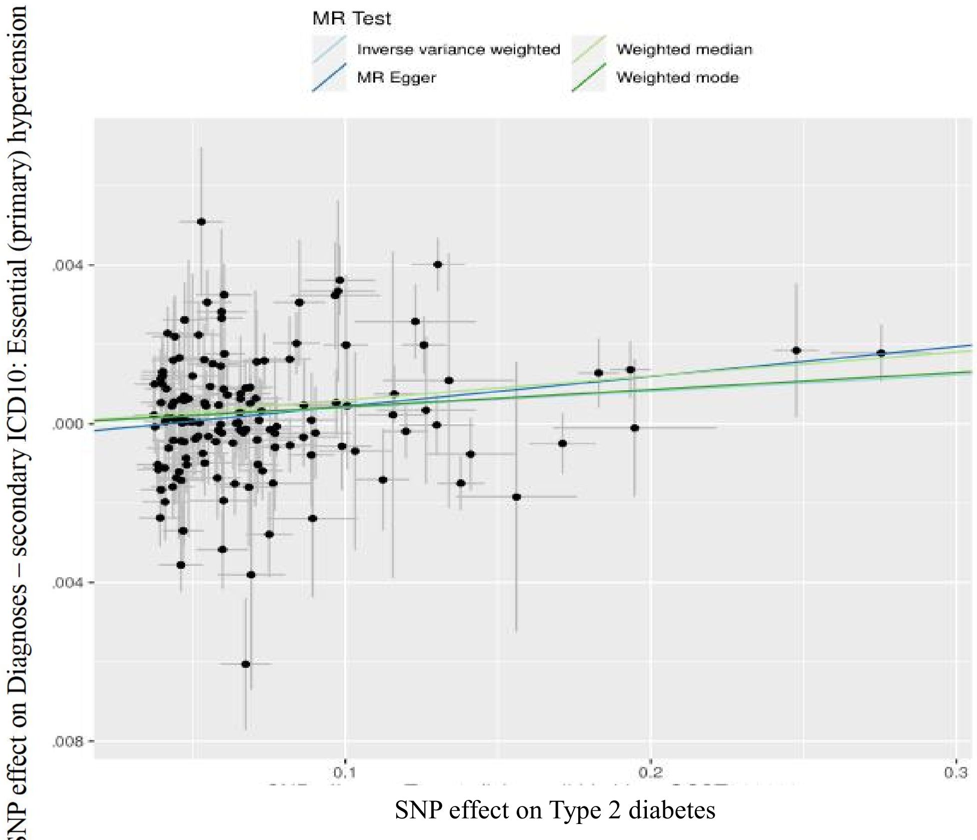
MR Test
Inverse variance weighted
MR Egger
Weighted median
Weighted mode
SNP effect on Diagnoses – secondary ICD10: Essential (primary) hypertension
.004
.000
.004
.008
0.1
0.2
0.3
SNP effect on Type 2 diabetes

(C)

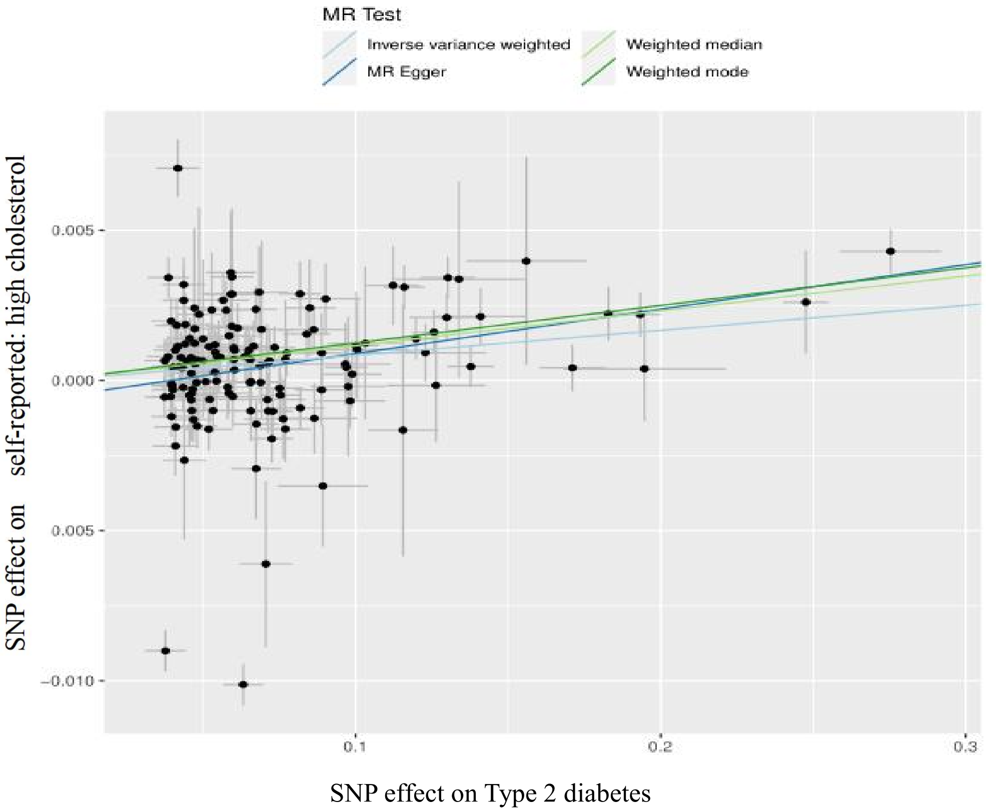
MR Test
Inverse variance weighted
MR Egger
Weighted median
Weighted mode
SNP effect on self-reported: high cholesterol
0.005
0.000
0.005
−0.010
0.1
0.2
0.3
SNP effect on Type 2 diabetes

(D)

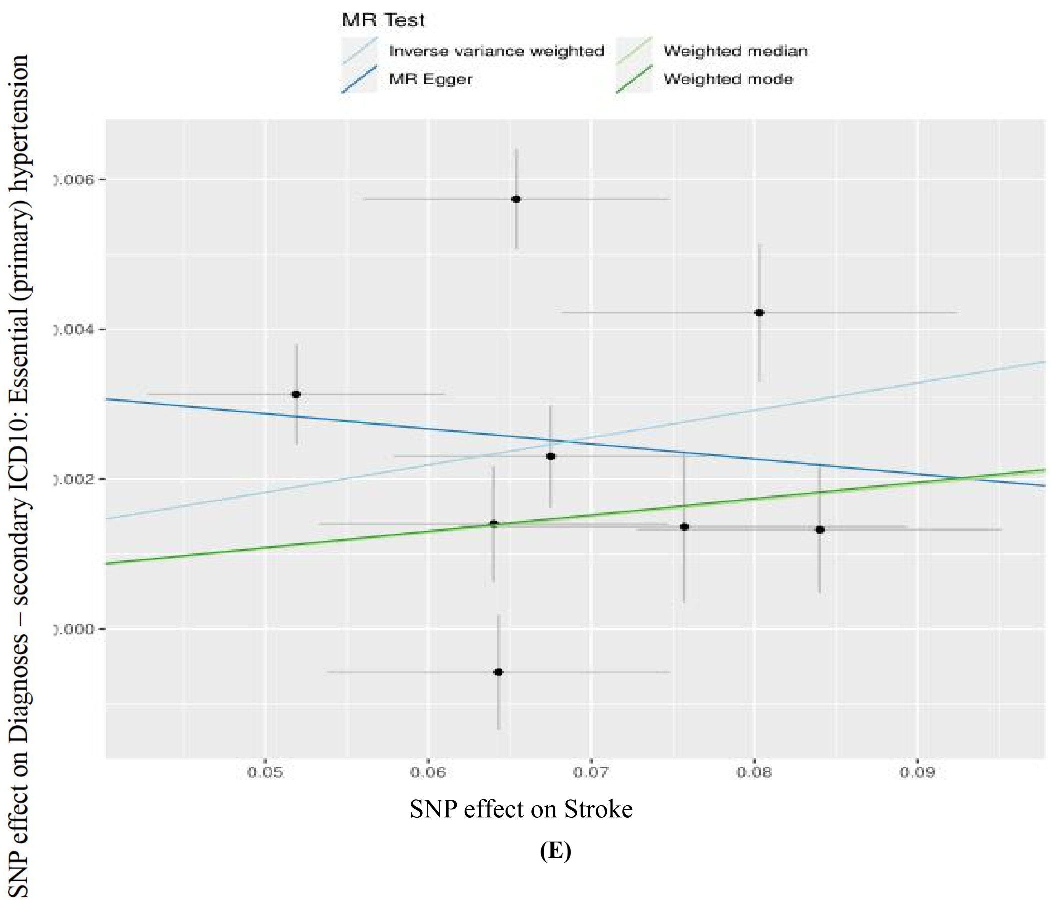
MR Test
Inverse variance weighted
MR Egger
Weighted median
Weighted mode
SNP effect on Diagnoses – secondary ICD10: Essential (primary) hypertension
0.006
0.004
0.002
0.000
0.05
0.06
0.07
0.08
0.09
SNP effect on Stroke

(E)

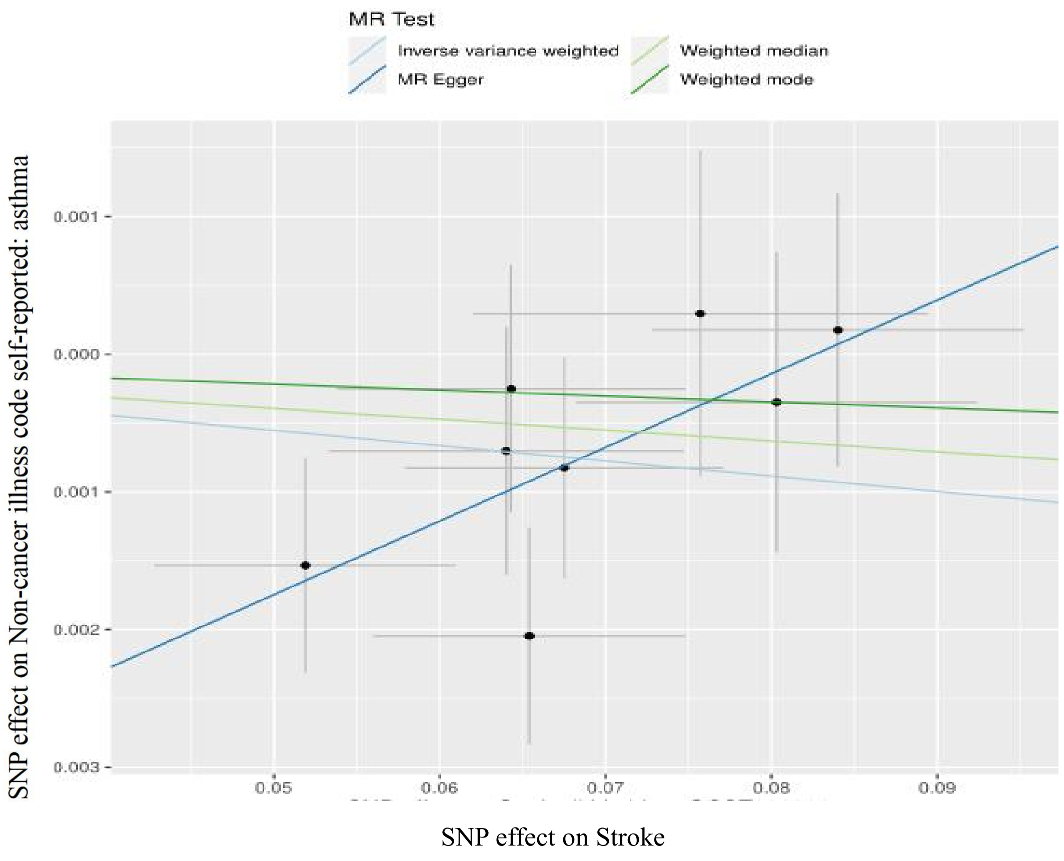
MR Test
Inverse variance weighted
MR Egger
Weighted median
Weighted mode
SNP effect on Non-cancer illness code self-reported: asthma
0.001
0.000
0.001
0.002
0.003
0.05
0.06
0.07
0.08
0.09
SNP effect on Stroke

(F)

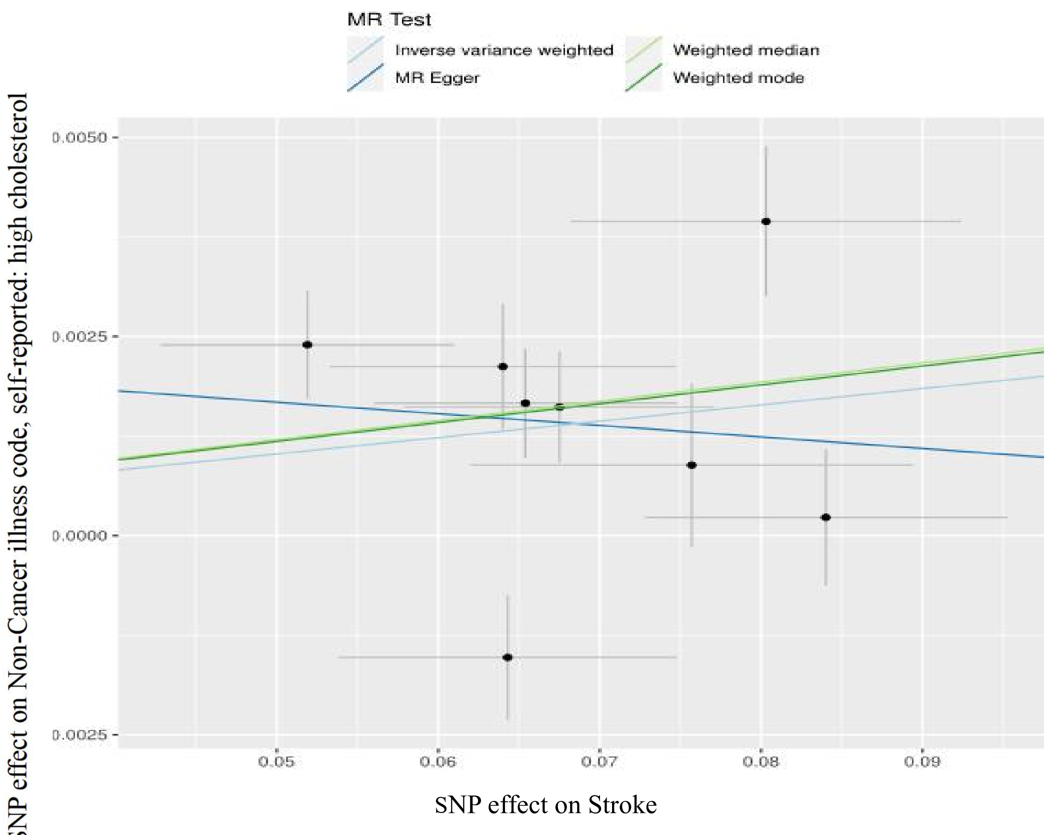
MR Test
Inverse variance weighted
MR Egger
Weighted median
Weighted mode
SNP effect on Non-Cancer illness code, self-reported: high cholesterol
0.0050
0.0025
0.0000
0.0025
0.05
0.06
0.07
0.08
0.09
SNP effect on Stroke

(G)

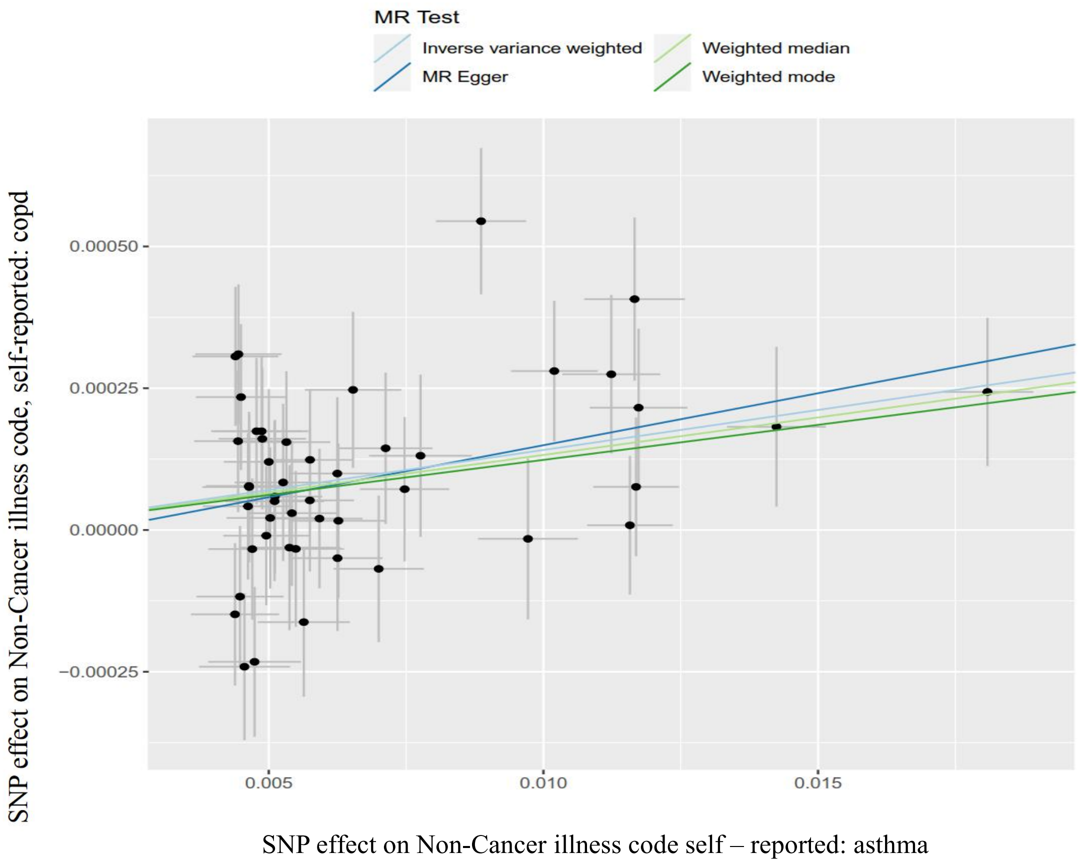
MR Test
Inverse variance weighted
MR Egger
Weighted median
Weighted mode
SNP effect on Non-Cancer illness code, self-reported: copd
0.00050
0.00025
0.00000
−0.00025
0.005
0.010
0.015
SNP effect on Non-Cancer illness code self – reported: asthma

(H)

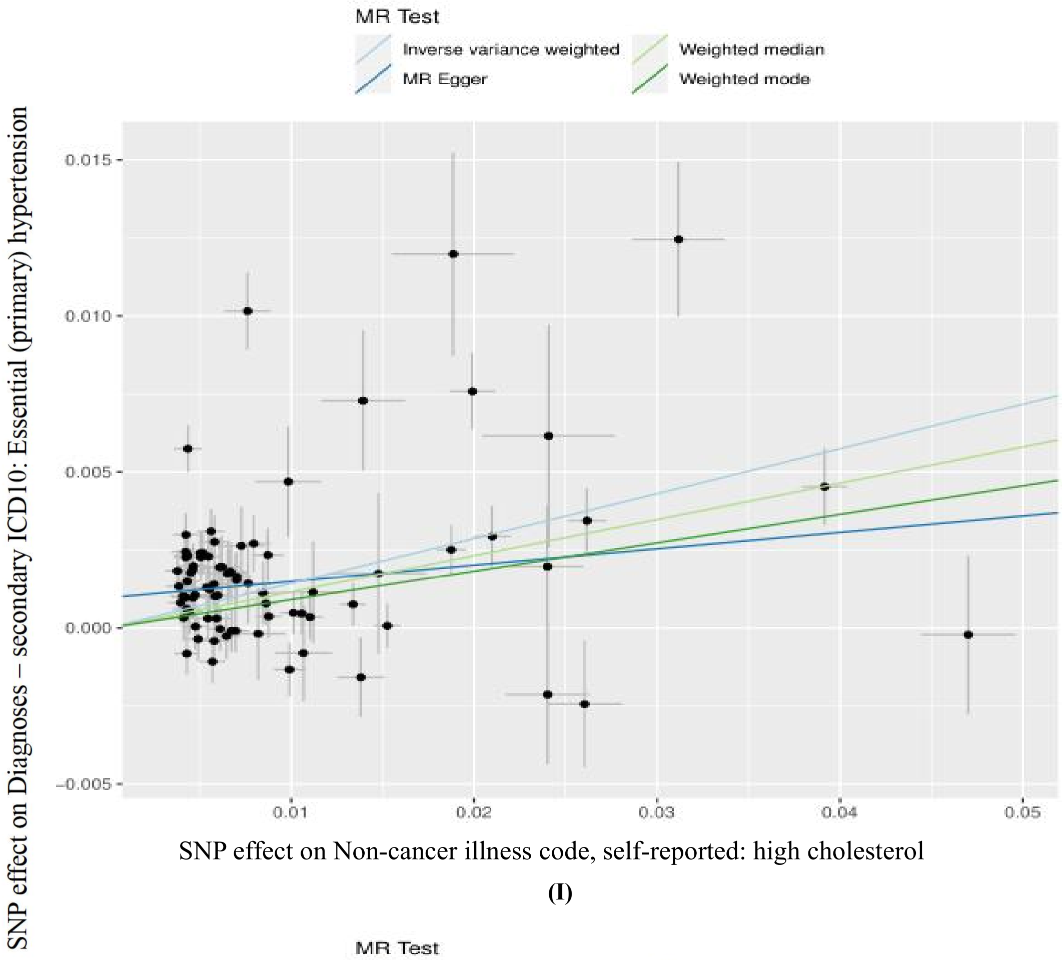
MR Test
Inverse variance weighted
MR Egger
Weighted median
Weighted mode
0.015
0.010
0.005
0.000
−0.005
0.01
0.02
0.03
0.04
0.05
SNP effect on Diagnoses – secondary ICD10: Essential (primary) hypertension
SNP effect on Non-cancer illness code, self-reported: high cholesterol

(I)

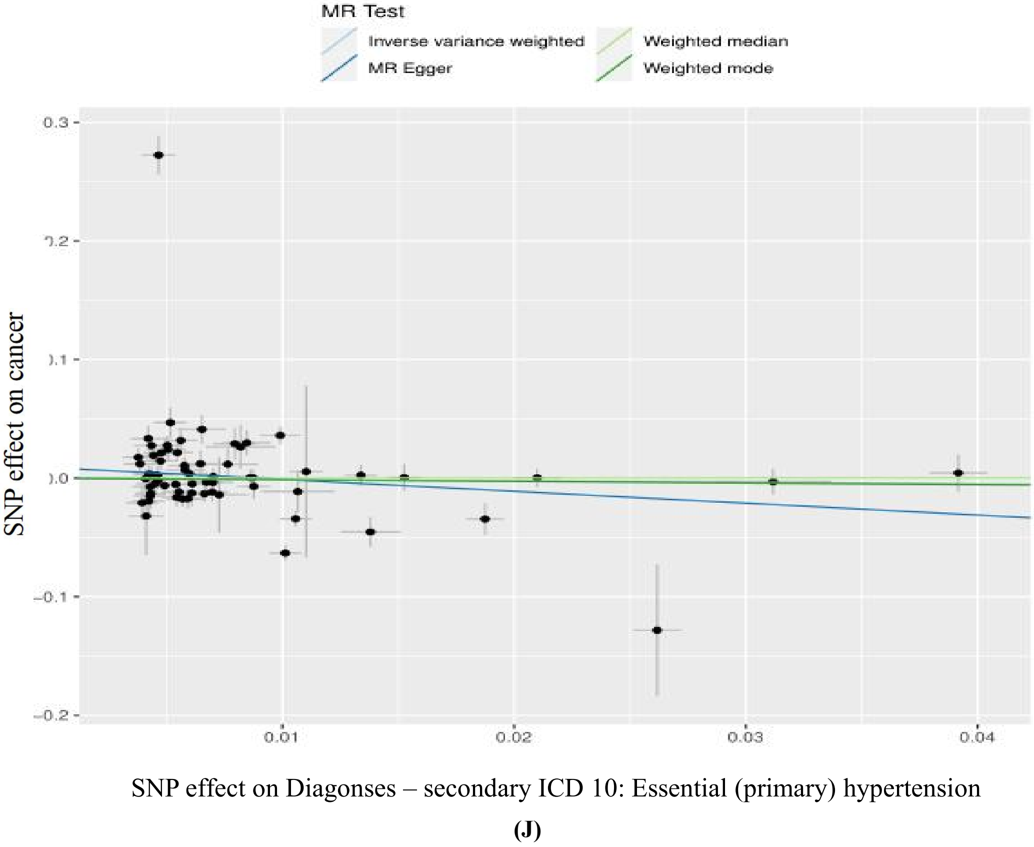
MR Test
Inverse variance weighted
MR Egger
Weighted median
Weighted mode
0.3
0.0
−0.1
−0.2
0.01
0.02
0.03
0.04
SNP effect on cancer
SNP effect on Diagonses – secondary ICD 10: Essential (primary) hypertension

(J)

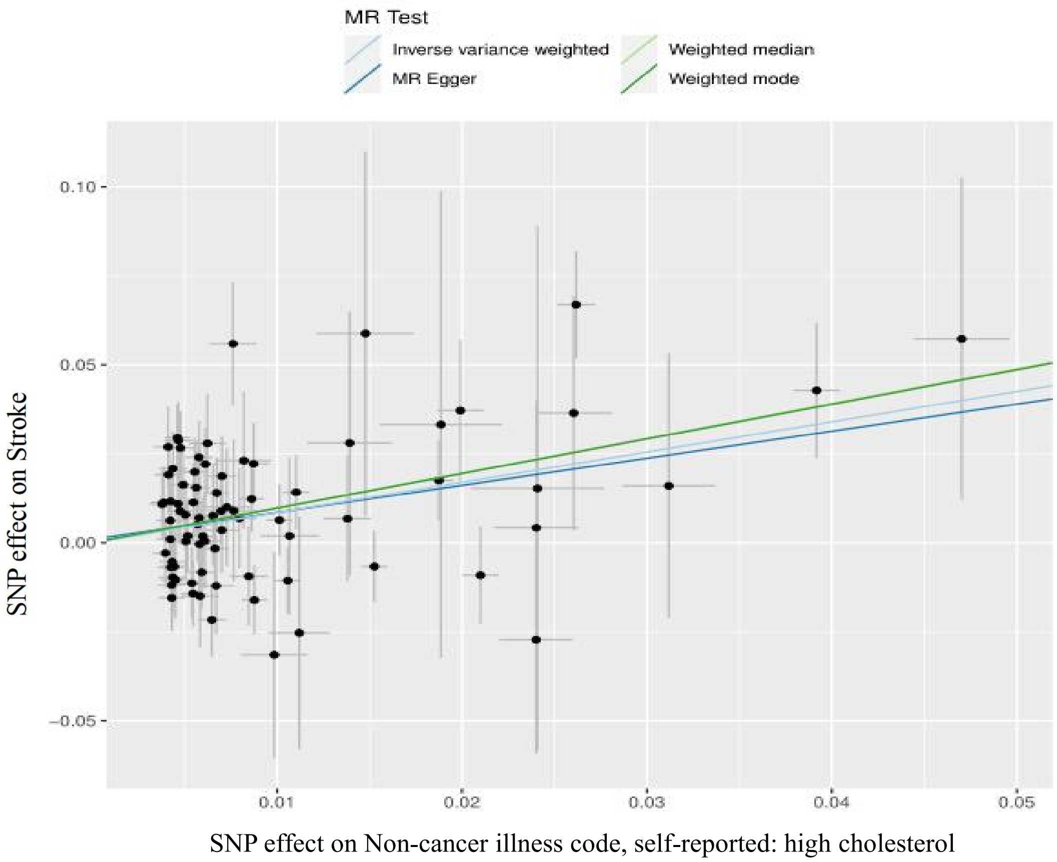
MR Test
Inverse variance weighted
MR Egger
Weighted median
Weighted mode
SNP effect on Stroke
0.10
0.05
0.00
−0.05
0.01
0.02
0.03
0.04
0.05
SNP effect on Non-cancer illness code, self-reported: high cholesterol

(K)

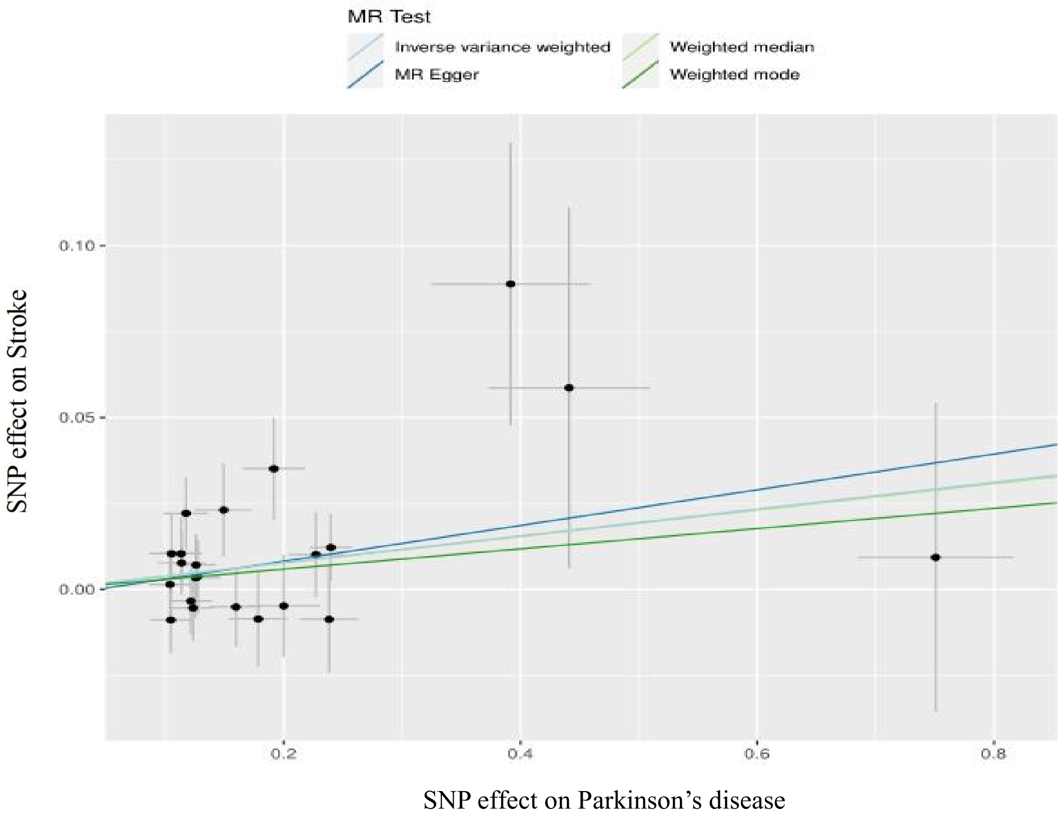
MR Test
Inverse variance weighted
MR Egger
Weighted median
Weighted mode
SNP effect on Stroke
0.10
0.05
0.00
0.2
0.4
0.6
0.8
SNP effect on Parkinson's disease

(L)

S7 Fig. Scatter plots of estimated causal associations with significant significance for 14 pairs of phenotypes. (IVW<0.05). The different colored lines represent the different MR analysis methods, respectively. The horizontal coordinates show the exposure variables and the vertical coordinates show the outcome variables. (**A**) Hypertension against stroke risk; (**B**)Hypertension against high cholesterol; (**C**) Type 2 diabetes against hypertension risk; (**D**)Type 2 diabetes against high cholesterol; (**E**)Stroke against hypertension risk; (**F**) Stroke against asthma. (**G**) Stroke against high cholesterol. (**H**) Asthma against chronic obstructive pulmonary disease. (**I**) High cholesterol against hypertension. (**J**) Hypertension against cancer. (**K**) High cholesterol against stroke. (**L**) Parkinson's disease against stroke. The slopes of each line represent the causal association for each method.
